# Unified Modulation Matrix-Based Shared Control for Teleoperated Multi-Robot Formation and Obstacle Avoidance

**DOI:** 10.3390/s26082387

**Published:** 2026-04-13

**Authors:** Ruidong Chen, Zhuoyue Zhang, Zhiyao Zhang, Jinyan Li, Haochen Zhang

**Affiliations:** School of Automation and Electrical Engineering, Lanzhou University of Technology, Lanzhou 730050, China; 230052201004@lut.edu.cn (R.C.);

**Keywords:** teleoperation, intent-mediated, shared control, multi-robot formation, asymmetric vortex field, obstacle avoidance

## Abstract

Multi-omnidirectional mobile robot formations offer significant advantages for applications in unstructured environments. However, under constraints such as limited field of view and high operator cognitive load, existing teleoperation frameworks struggle to guarantee formation safety and stability. In this study, a bilateral shared control framework for multi-robot formation that integrates intent perception and vortex-field modulation is proposed. First, an Intent-Mediated Asymmetric Vortex Modulation (IM-AVM) strategy is developed, where the operator’s micro-intentions are mapped to determine the topological orientation of a vortex field. By constructing a dynamic asymmetric modulation matrix, saddle points in the potential field are geometrically eliminated, enabling deadlock-free obstacle avoidance while maintaining a rigid formation. Second, a multi-dimensional perception-based dynamic authority arbitration and topological deadlock escape mechanism is constructed, facilitating a seamless transition from assisted deadlock to autonomous escape. Finally, a formation coordination system based on anisotropic flow field modulation and adaptive sliding mode control is designed. Rigid formation constraints are transformed into a tangential safe flow field, and robust tracking is subsequently achieved through an Adaptive Nonsingular Fast Terminal Sliding Mode Controller (ANFTSMC). Theoretical analysis and experimental results demonstrate that the proposed framework achieves collision-free navigation for the formation in simulated environments.

## 1. Introduction

Driven by the demand for complex remote tasks, multi-robot systems have evolved from standalone units into highly coordinated swarms [1,2,3,4,5]. The slave end can flexibly adapt to various operational scenarios through multi-mobile robot formation control [6]. However, restricted fields of view and high operator cognitive load often compromise formation safety and stability in unstructured environments [7,8,9].

In typical formation teleoperation scenarios, several architectures are prevalent, including the leader-follower approach [10], behavior-based methods [11], the virtual structure method [12], and potential function-based methods [13]. The leader-follower structure is widely adopted, as collective behavior is governed directly by the leader’s motion [14], and followers can acquire the leader’s state via distributed observers [15]. Recent advances have further extended this to collisions-free distributed cooperative tracking for nonlinear MASs, even under communication constraints such as limited data rates [16]. The system adopts the leader-follower approach, which aims to combine human global judgment with robotic execution precision. However, classic teleoperation only provides a narrow two-dimensional field of view and lacks haptic perception of the environment. This disconnection between perception and execution can lead to formation collisions, disintegration, or entrapment in environmental deadlocks [17,18].

Most existing work primarily addresses individual issues in multi-agent teleoperation control, such as obstacle avoidance methods for dealing with obstacles [19,20,21], advanced control techniques (e.g., predictive control [22], adaptive control [23,24,25] etc.) for handling nonlinearities and various uncertainties, and formation control for managing multi-agent movements [10,12]. However, few studies address integrated challenges in unstructured scenarios. Traditional Artificial Potential Field (APF) methods [26,27,28,29], though widely adopted for their reactive safety, often suffer from local minima and computational delays [30].

Specifically, Rodríguez-Seda et al. [10] proposed a PD-based formation control method that utilizes APF for obstacle avoidance, but this approach may induce wave reflection phenomena due to signal transformations and fails to achieve satisfactory tracking performance. Franchi et al. [2] proposed a distributed control scheme based on the leader-follower model, yet it imposes excessive constraints on the formation during obstacle avoidance in cluttered environments. Additionally, numerous other methods, [31,32] have been developed for multi-agent teleoperation, but some lack obstacle avoidance functionality, others do not account for nonlinearities and various uncertainties, and most teleoperation approaches only consider the scenario with a single agent at the slave end.

In U-shaped or dense obstacles, APF-based methods frequently stagnate at non-target saddle points, known as local minima. Existing studies [33] typically introduce disturbances or fixed bias forces based on static heuristics to break symmetry. However, traditional fixed-bias algorithms often deviate from the operator’s commands during specific obstacle avoidance maneuvers. While some multi-layer frameworks address local minima through leader selection [34], they often overlook the geometric conflicts between formation constraints and unstructured environments [35]. Most obstacle avoidance algorithms simplify robots as point masses [36], neglecting the formation’s macroscopic footprint and motion constraints [37]. Consequently, the core challenge remains planning continuous trajectories that conform to environmental topology while strictly maintaining formation integrity.

Shared autonomy enhances obstacle avoidance but limits human control authority over robots [38]. Some systems mitigate risk by augmenting known paths [39] or predicting human intent for parallel autonomy [40]. Force-reflective shared autonomy [28] allows the human operator to maintain full control during teleoperation while providing collision avoidance assistance. Cooperative force-guidance systems must assist users in obstacle avoidance for navigation toward the target. To minimize the cognitive load on human users, the system should apply force feedback only when necessary. In bilateral teleoperation under communication delays, existing research has introduced finite-time proportional damping injection and event-triggered communication mechanisms to address latency [23,25,41]. While these methods effectively ensure system stability, they inherently attenuate the force feedback signals from the slave side, thereby degrading the transparency of haptic interaction.

To address the aforementioned issues, this study proposes a bilateral shared control framework for multi-robot formation that integrates intent perception and vortex-field modulation. The main contributions of this research are summarized as follows:

(1) An Intent-Mediated Asymmetric Vortex Modulation (IM-AVM) strategy is proposed to resolve the local minima and saddle-point deadlocks in teleoperation. By mapping operator micro-intentions to flow-field chirality, this approach geometrically eliminates potential field local minima while maintaining high haptic transparency.
(2) A multi-dimensional situational awareness and deadlock escape mechanism is established to address the entrapment issues in complex U-shaped obstacles. By integrating geometric, physical, and interaction conflict indices, the system achieves a seamless transition from assisted deadlock to autonomous escape via a maximum flux search algorithm.
(3) An adaptive formation coordination system based on anisotropic flow-field modulation is developed to resolve the conflict between rigid formation constraints and narrow environmental gaps. This system utilizes a dynamic scaling factor σ and an adaptive compliance gain $k_{p,i}^{*}$ to ensure robust, collision-free navigation under lumped uncertainties. The remainder of this paper is organized as follows: In Section 2, the dynamic model of the omnidirectional mobile robot formation and the bilateral teleoperation framework are established. Section 3 elaborates on the design principles of the IM-AVM strategy and the hierarchical controller. In Section 4, rigorous proofs of system stability and convergence are provided based on Lyapunov theory. Section 5 validates the effectiveness of the proposed method through experimental analysis and discussion.

## 2. System Modeling and Environmental Representation

This section aims to transform physical constraints into mathematically tractable formulations for control algorithm development. First, the kinematic equations of the Mecanum-wheeled omnidirectional platform are derived, and the uncertainty bounds are analyzed to identify the disturbance characteristics that need to be suppressed by the Adaptive Nonlinear Fast Terminal Sliding Mode Controller (ANFTSMC). Second, the frequency-domain origins of passivity degradation caused by communication delays are examined, providing a theoretical foundation for predictive damping injection at the interaction layer. Third, an anisotropic generalized distance field incorporating virtual linkages is constructed. Finally, a relative pose model for formation control is established to unify the multi-objective control requirements.

### 2.1. Modeling of the Mecanum-Wheeled Omnidirectional Mobile Robot

#### 2.1.1. Kinematic Model

The global inertial coordinate frame Σ***_I_*** and the robot body-fixed frame Σ***_R_*** are defined. The pose vector of an arbitrary robot in Σ***_I_*** is given by: $q={\left[x,y,\theta \right]}^{T}$. Its velocity expressed in Σ***_R_*** is: $v={\left[v_{x},v_{y},\omega \right]}^{T}$.

The relationship between these two representations is governed by the following rotational transformation:

(1) $$\dot{q}=R(\theta )v,$$

where the rotation matrix R(θ) is defined as:

(2) $$R(\theta )=\left[\begin{array}{ccc} \cos\theta_{i} & -\sin\theta_{i} & 0\\ \sin\theta_{i} & \cos\theta_{i} & 0\\ 0 & 0 & 1 \end{array}\right].$$

An omnidirectional mobile platform equipped with Mecanum wheels is adopted in this study. The inverse kinematic relationship between the vehicle velocity and the angular velocities of its four wheels is given by:

(3) $$\omega =\frac{1}{r}H\cdot v_{b},$$

where $\omega ={\left[\omega_{1},\omega_{2},\omega_{3},\omega_{4}\right]}^{T}$ is the vector of wheel angular velocities; $v_{b}={\left[v_{x},v_{y},\omega \right]}^{T}$ is the velocity vector (longitudinal, lateral, and yaw rates) in the body-fixed frame Σ***_R_***; *r* is the wheel radius; and $H\in {\mathbb{R}}^{4\times 3}$ is the inverse Jacobian matrix, defined as:

(4) $$H=\left[\begin{array}{ccc} 1 & -1 & -(l_{x}+l_{y})\\ 1 & 1 & \left(l_{x}+l_{y}\right)\\ 1 & 1 & -(l_{x}+l_{y})\\ 1 & -1 & \left(l_{x}+l_{y}\right) \end{array}\right].$$

Here, *l_x_* and *l_y_* represent the half-track width and half-wheelbase, respectively. Equation (3) indicates that precise control of the vehicle omnidirectional motion can be achieved by solving for the required individual wheel speeds.

#### 2.1.2. Dynamic Model

However, a kinematic model alone is insufficient to describe the dynamic response of the robot caused by factors such as mass distribution and ground friction. Therefore, a second-order dynamic model of the robot is established, considering simulated nonlinear friction, load variations, and inertial effects:

(5) $$M(q)\overset{..}{q}+C(q,\dot{q})\dot{q}+D\dot{q}+\tau_{d}=\tau ,$$

where $M\in {\mathbb{R}}^{3\times 3}$ is the symmetric positive definite inertia matrix; C is the Coriolis and centrifugal matrix; D is the viscous friction damping matrix; ***τ****_d_* represents stochastic disturbances injected into the HIL platform to emulate the uncertainties of real-world unstructured environments; and ***τ*** is the generalized control torque vector.

To facilitate subsequent controller design, the model is transformed into a standard state-space form:

(6) $$\overset{..}{q}=M^{-1}(\tau -C\dot{q}-D\dot{q})-M^{-1}\tau_{d}.$$

Here, **u** = ***τ*** is the control input to be designed, and $\Delta =-M^{-1}\tau_{d}$ is the lumped uncertainty term, which is assumed to be bounded.

### 2.2. Modeling of the Bilateral Teleoperation System

**Figure 1:**
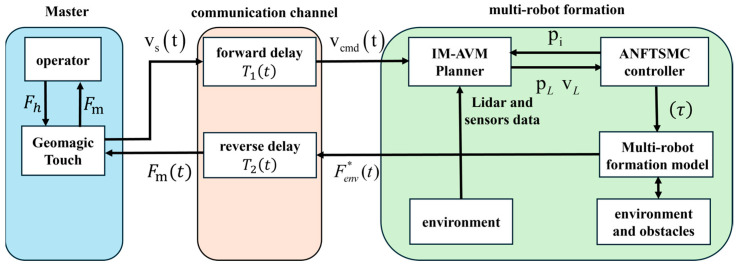
Depiction of the bilateral teleoperation framework.

As illustrated in Figure 1, the constructed bilateral teleoperation multi-robot formation control system primarily consists of three components:

The master-side interaction subsystem, the bidirectional communication channel with time delays, and the slave-side formation control subsystem. Motion commands *v_m_*(*t*) generated at the master side are transmitted to the slave side via the forward channel, while the environmental interaction forces ***F****_env_*(*t*) perceived at the slave side are transmitted back to the master side through the reverse channel, providing force feedback to the operator.

#### 2.2.1. Kinematic Mapping and Signal Preprocessing

**Figure 2:**
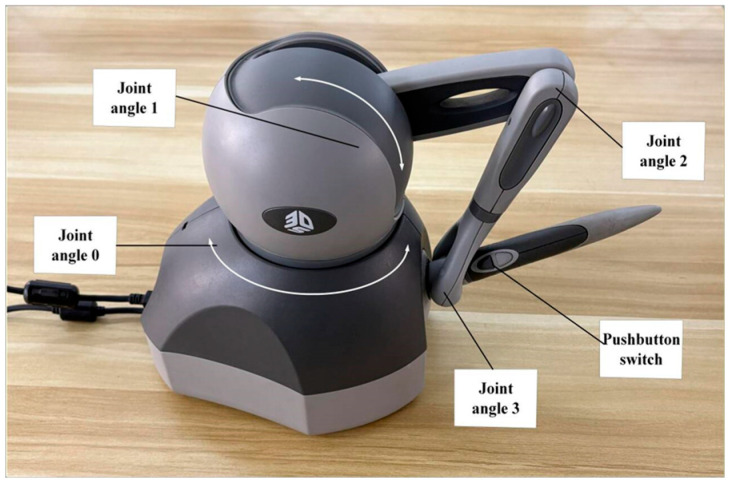
Geomagic Touch.

A Geomagic Touch haptic device is utilized as the master-side interaction interface in this study. Its physical structure is a serial articulated mechanism, as shown in Figure 2. The device primarily consists of four rotary joints, a stylus, and a dual-button switch.

In this study, only the waist joint (*q*_0_) and the shoulder joint (*q*_1_) of the haptic device are utilized for low-dimensional control, while the remaining joints are kept in a locked state. The raw joint vector is defined as $q={\left[q_{0},q_{1}\right]}^{T}\in {\mathbb{R}}^{2}$. To suppress sensor zero-drift and physiological tremor, the input signals are normalized using a dead-zone operator:

(7) $$q_{eff}=\Phi (q,\delta )=\left[\begin{array}{c} \phi (q_{0},\delta_{w})\\ \phi (q_{1},\delta_{s}) \end{array}\right],\;\;\;\;\mathrm{where}\;\phi (q,\delta )=\left\{\begin{array}{ll} q-\mathrm{sgn}(q)\delta , & |q|>\delta \\ 0, & |q|\leq \delta \end{array}\right.\;.$$

Here, $q_{eff}={\left[q_{0},q_{1}\right]}^{T}$ is the effective joint vector, Φ represents the nonlinear dead-zone operator, and *δ* is the hysteresis threshold. Subsequently, a forward kinematic mapping $F(q_{eff})$ is established from the joint space Q to the Cartesian task space $X\subset {\mathbb{R}}^{2}$, yielding the velocity mapping relationship:

(8) $${\dot{x}}_{m}=\left[\begin{array}{c} {\dot{x}}_{m}\\ {\dot{y}}_{m} \end{array}\right]\approx \underset{J_{0}}{\underset{︸}{\left[\begin{array}{cc} \frac{\partial x}{\partial q_{0}} & \frac{\partial x}{\partial q_{1}}\\ \frac{\partial y}{\partial q_{0}} & \frac{\partial y}{\partial q_{1}} \end{array}\right]{|}_{q=0}}}\left[\begin{array}{c} {\dot{q}}_{0}\\ {\dot{q}}_{1} \end{array}\right]=\left[\begin{array}{cc} l_{1} & 0\\ 0 & l_{2} \end{array}\right]\left[\begin{array}{c} {\dot{q}}_{0}\\ {\dot{q}}_{1} \end{array}\right],$$

where $J_{0}$ is the decoupled constant Jacobian matrix, and $l_{1},l_{2}$ are the equivalent lever arm lengths for the waist and shoulder joints, respectively. Equation (8) indicates that the Waist joint (*q*_0_) maps to the lateral velocity ${\dot{x}}_{m}$, and the Shoulder joint (*q*_1_) maps to the longitudinal velocity ${\dot{y}}_{m}$.

#### 2.2.2. Master-Side Dynamic Model

To characterize the force perception properties of the human–robot interaction, the dynamic equation of the master device is established:

(9) $$M(x_{m}){\overset{..}{x}}_{m}+C(x_{m},{\dot{x}}_{m}){\dot{x}}_{m}+g(x_{m})=f_{h}+f_{m}.$$

Neglecting the Coriolis and gravity terms and linearizing the nonlinear inertia matrix $M(x_{m})$ around the operating point yields an impedance model:

(10) $$\underset{M_{m}}{\underset{︸}{\left[\begin{array}{cc} m_{x} & 0\\ 0 & m_{y} \end{array}\right]}}{\overset{..}{x}}_{m}+\underset{B_{m}}{\underset{︸}{\left[\begin{array}{cc} b_{x} & 0\\ 0 & b_{y} \end{array}\right]}}{\dot{x}}_{m}=f_{h}+f_{m},$$

where $M_{m}$ and $B_{m}$ are the equivalent inertia and physical damping matrices, respectively; $f_{h}$ is the driving force applied by the operator; and $f_{m}$ is the feedback force output by the haptic device.

#### 2.2.3. Velocity Mapping

To resolve the kinematic dissimilarity between the master and slave devices, a velocity control law based on the Jacobian transpose is constructed. Using the effective joint angles from Equation (7), the velocity command $v_{cmd}$ in the slave’s body-fixed frame $v_{cmd}$ is directly mapped as:

(11) $$v_{s}=\Gamma \cdot J_{0}\cdot q_{eff}=\left[\begin{array}{cc} \gamma_{x}l_{1} & 0\\ 0 & \gamma_{y}l_{2} \end{array}\right]\left[\begin{array}{c} q_{eff,1}\\ q_{eff,2} \end{array}\right],$$

where $\Gamma =\mathrm{diag}(\gamma_{x},\gamma_{y})$ is the adjustment matrix. Equation (11) indicates that $q_{0}$ independently controls the lateral translation $v_{x}$, and $q_{1}$ independently controls the longitudinal translation $v_{y}$, and $v_{s}$ is the slave reference velocity vector.

#### 2.2.4. Communication Channel Model

The forward time delay is denoted as $T_{f}(t)$, and the backward time delay as $T_{b}(t)$. The signal transmission characteristics of the system are described as follows:

(12) $$\left\{\begin{array}{l} v_{cmd}(t)=v_{s}\left(t-T_{f}(t)\right)\\ f_{m}(t)=\alpha \cdot f_{env}\left(t-T_{b}(t)\right) \end{array}\right.,$$

where α is the force feedback gain. The delay functions $T_{i}(t)$ are assumed to be continuously differentiable and bounded ($0\leq T_{i}(t)\leq T_{max}$), with their rates of change satisfying ${\dot{T}}_{i}(t)<1$. The delayed slave velocity command, denoted as $v_{cmd}(t)$, is obtained by applying the forward time delay $T_{f}(t)$ to the slave reference velocity $v_{s}$.

### 2.3. Enhanced Anisotropic Environmental Field

To address the challenge of obstacle avoidance for formation safety in unstructured environments containing complex obstacles, an enhanced potential field based on anisotropic distance is constructed.

#### 2.3.1. Anisotropic Generalized Distance Field

To accommodate obstacles with varying length-to-width ratios and to suppress unnecessary lateral avoidance maneuvers, the anisotropic Euclidean distance field between the robot position **x** and the *i*-*th* feature point $p_{i}$ belonging to the augmented obstacle set $P_{aug}$ is defined. First, the distance in a rotated and scaled frame is computed:

(13a) $$\begin{array}{l} D_{i}(x)=\sqrt{{\left(x-p_{i}\right)}^{T}R(\theta )\left[\begin{array}{cc} \frac{1}{\lambda_{long}^{2}} & 0\\ 0 & \frac{1}{\lambda_{lat}^{2}} \end{array}\right]R{\left(\theta \right)}^{T}(x-p_{i})} \end{array},$$

(13b) $$n_{i}(x)=\nabla D_{i}/||\nabla D_{i}||\;.$$

Here, $x,p_{i}\in {\mathbb{R}}^{2}$ are the robot’s current position vector and the coordinates of a discrete obstacle point, respectively; **R**(*θ*) is the robot’s rotation matrix; $\lambda_{long}$ and $\lambda_{lat}$ are the longitudinal and lateral sensitivity radii, respectively. By setting $\lambda_{long}>\lambda_{lat}$, this metric generates elliptical isolines elongated along the robot’s heading direction in the Cartesian space, causing the repulsive influence of obstacles to extend much further longitudinally than laterally. The sensitivity radii $\lambda_{long}$ and $\lambda_{lat}$ are designed to be anisotropic., this configuration expands the leader’s perceptual envelope to encompass the formation-level physical footprint, ensuring that any ‘safe gap’ identified by the vortex modulation provides sufficient clearance for all followers.

#### 2.3.2. Virtual Linking Mechanism

When the distance between two discrete obstacle points is smaller than the minimum safe width required for the robot to pass through, the obstacle avoidance algorithm might erroneously identify it as a traversable gap, leading to oscillations or collisions in narrow passages. To address this, an obstacle connectivity enhancement mechanism is introduced.

The set of discrete obstacle points detected by the sensor is defined as:$P_{obs}=\{p_{1},p_{2},\ldots ,p_{m}\}$. For any two adjacent obstacle points $p_{j}$ and $p_{j+1}$, their Euclidean distance is first calculated as: $d_{j,j+1}=\|p_{j+1}-p_{j}\|$. If $d_{j,j+1}<W_{safe}$, the region is deemed impassable. Subsequently, a set of virtual points $P_{virt}$ is generated via linear interpolation to close this gap:

(14) $$p_{virt}^{\left(k\right)}=p_{j}+\frac{k}{N_{step}}\left(p_{j+1}-p_{j}\right),\;\;\;\;k=1,2,\ldots ,N_{step},$$

where $p_{virt}^{\left(k\right)}\in {\mathbb{R}}^{2}$ represents the position vector of the *k*-th virtual filling point; $p_{j},p_{j+1}\in {\mathbb{R}}^{2}$ are the original obstacle boundary points; the interpolation density factor is given by $N_{step}=d_{j,j+1}/\delta_{res}$, and $W_{safe}$ is the critical expansion threshold, typically chosen to be larger than the robot chassis diameter.

By incorporating the virtual point set $P_{virt}$ into the original obstacle set, the augmented set is obtained as $P_{aug}=P_{obs}\cup P_{virt}$. This approach topologically closes all physically infeasible narrow passages.

#### 2.3.3. Vectorized Representation of Environmental Constraints

Unlike traditional APF methods that directly superimpose repulsive forces, this work utilizes the negative gradient information of the enhanced potential field to characterize the geometric repulsion exerted by the environment on the robot. The aggregate repulsive potential field function is defined as $U_{rep}(x)$, and the corresponding constraint interaction vector $F_{env}(x)$ is derived as follows:

(15) $$F_{env}(x)=-\nabla U_{rep}(x)=\sum_{i\in P_{aug}}\eta \left(\frac{1}{D_{i}(x)}-\frac{1}{\rho_{0}}\right)\frac{1}{D_{i}^{2}(x)}\cdot n_{i}(x),$$

where *η* is the gain coefficient, and $\rho_{0}$ defines the range of influence.

### 2.4. Leader-Follower Formation Dynamic Geometric Modeling

A multi-robot formation consisting of n followers and one leader is considered in this study. Unlike traditional rigid formation models, a dynamic scaling factor is introduced here, allowing the formation to autonomously compress its configuration in narrow spaces.

#### 2.4.1. Formation Transformation and Relative Pose Description

The state vector of the leader in the global coordinate frame is defined as $q_{L}={\left[x_{L},y_{L},\theta_{L}\right]}^{T}$, and the actual state of the *i*-th follower is defined as: $q_{i}={\left[x_{i},y_{i},\theta_{i}\right]}^{T}$. To describe the desired geometric relationship of the follower relative to the leader, a predefined nominal geometric offset is defined as: $l_{i0}={\left[l_{ix},l_{iy}\right]}^{T}$. Considering the dynamic changes in environmental constraints, a time-varying scaling factor σ(t)∈(0,1] is introduced. The desired position of the follower in the global coordinate frame is constructed as:

(16) $$p_{id}(t)=p_{L}(t)+R(\theta_{L})\Lambda (t)l_{i0},$$

where **p***_L_* = [x*_L_*,y*_L_*]*^T^* is the leader’s position, **R**(*θ*_L_) is the rotation matrix, and Λ(t)=diag(1,σ(t)) is the deformation matrix. Equation (16) indicates that the formation is elastically compressed only in the lateral (*y*-axis) direction, while the longitudinal spacing remains constant.

#### 2.4.2. Kinematic Feedforward and Reference Velocity Generation

To achieve high-precision formation keeping, the follower’s control objective is not only to eliminate position errors but also to match in real-time the transport velocity induced by the leader’s motion. Differentiating Equation (16) and neglecting the transient rate of change in the scaling factor (assuming $\dot{\sigma }\approx 0$), the theoretical feed forward velocity for the follower is obtained:

(17) $$v_{ff,i}=v_{L}+\omega_{L}SR(\theta_{L})\Lambda l_{i0},$$

where $v_{L}$ and $\omega_{L}$ are the linear and angular velocities of the leader, respectively, and S=[0,−1;1,0] is the rotation operator.

**Remark 1**.  *In the derivation of the feedforward velocity in Equation (17), the time derivative of the scaling factor*$\dot{\sigma }$*is not explicitly compensated. This simplification is justified by the time-scale separation between the formation scaling evolution and the high-frequency control loop (20 Hz). Specifically, the scaling factor is processed with a low-pass filter (with smoothing factor* β=0.3*), ensuring that* $\dot{\sigma }$ *remains a small-scale term. Any residual tracking error caused by this approximation is treated as a lumped kinematic disturbance and is effectively suppressed by the robust integral-type sliding surface of the ANFTSM.*

The nominal reference velocity $v_{raw,i}$ for the follower is defined as a linear combination of the feedforward term and a position error feedback term:

(18) $$v_{raw,i}=v_{ff,i}+k_{p}(p_{id}-p_{i}),$$

where $k_{p}$ is the position error gain. This section has completed the mathematical modeling work. The kinematic and dynamic equations of the Mecanum-wheeled robot were derived; a bilateral teleoperation model incorporating time delays was established; an enhanced anisotropic environmental field was constructed to address the quantification of unstructured environments; and finally, the geometric description of the multi-robot formation was provided.

## 3. Shared Control and Formation Obstacle Avoidance Strategy for Teleoperation

**Figure 3:**
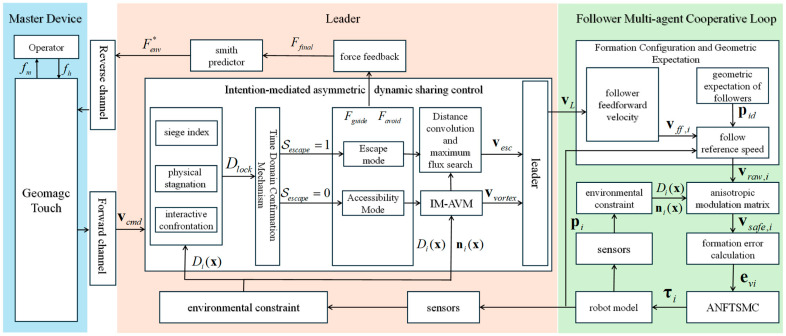
Architecture of the Hierarchical Collaborative Teleoperation Control System.

Addressing the coupled challenges of uncertain communication delays, environmental complexity, and rigid formation constraints in multi-robot formation teleoperation within unstructured environments, this section proposes a shared control architecture that integrates operator intent with autonomous obstacle avoidance mechanisms. The proposed architecture is illustrated in Figure 3.

In this study, a bilateral teleoperation and multi-robot cooperative control architecture is proposed. For the leader robot serving as the human–robot interaction interface, a bilateral shared control mechanism integrating Intent-Mediated Asymmetric Vortex Modulation (IM-AVM) and predictive damping injection is constructed. A dynamic arbitration strategy based on multi-dimensional perception is incorporated into this mechanism. By utilizing the topological chirality of the flow field, saddle points in the potential field are eliminated. Active dissipation of time-delay-induced energy is achieved through a Smith predictor, ensuring closed-loop passivity and stability. Upon detecting a U-shaped trap, an autonomous escape mode is triggered based on indices such as geometric entrapment and physical stagnation, whereby a locally optimal escape path is planned. For the follower robots in the formation, a collaborative system combining kinematic flow field modulation and robust dynamic tracking is designed. Rigid formation constraints are transformed into a tangential safe flow field compliant with the environment using an anisotropic flow field modulation strategy, enabling adaptive and compliant formation deformation. Robust tracking of the planned trajectories for the formation system is ensured by an Adaptive Nonsingular Fast Terminal Sliding Mode Controller (ANFTSMC).

### 3.1. Intent-Mediated Asymmetric Dynamic Shared Control

The leader robot, serving as the direct physical interface for human–robot interaction, is subject to operational lag caused by forward time delay *T*_1_ and force feedback distortion induced by backward time delay *T*_2_. A dual-loop compensation mechanism integrating micro-scale flow field modulation and macro-scale dynamic arbitration is designed in this section.

Traditional APF methods are prone to saddle point deadlocks when confronted with symmetric obstacles. To generate a deadlock-free obstacle avoidance guidance vector $v_{vortex}$, the concept of topological chirality is introduced. The rotational direction of the flow field is dynamically determined by the operator micro-scale intent, thereby breaking the symmetry of the potential field. Based on Equation (13b), an intent-mediated chirality factor utilizing hysteresis logic is designed in this study, which eliminates saddle points while avoiding numerical singularities and chattering:

(19) $$k_{chi}(t)=\left\{\begin{array}{ll} \mathrm{sgn}((n\times v_{cmd})\cdot e_{z}), & \mathrm{if}\;|(n\times v_{cmd})\cdot e_{z}|>\delta_{hyst}\\ k_{chi}(t-1), & \mathrm{otherwise} \end{array}\right.,$$

where *δ_hyst_* is the hysteresis threshold, and **e**_z_ is the unit vector along the vertical axis, this threshold is empirically set to $\delta_{hyst}=0.15$. Under noisy conditions near the decision boundary, it prevents the chirality factor $k_{chi}$ from rapidly oscillating between +1 and −1. This mechanism leverages the temporal continuity of human intent, inheriting the topological detour direction from the previous time step in critical states. Consequently, not only are saddle point deadlocks eliminated, but the non-singularity of the tangent vector basis is also guaranteed. Based on this, an intent-compliant chiral tangent vector $t_{chi}$ is constructed:

(20) $$t_{chi}=n\times (k_{chi}\cdot e_{z}).$$

This tangent vector ensures that the generated flow field topology consistently aligns with the operator intuition, preventing cognitive conflicts. To achieve a smooth transition from free space to constrained space, a dynamic modulation matrix **M**(**p**) based on eigenvalue decomposition is constructed:

(21) $$M(p)=E\Lambda E^{-1}=[n,t_{chi}]\left[\begin{array}{cc} \lambda_{n} & 0\\ 0 & \lambda_{t} \end{array}\right]{\left[n,t_{chi}\right]}^{-1}.$$

Here, λ_n_ is the normal damping eigenvalue: when approaching an obstacle, λ_n_ < 1, forcing the dissipation of normal collision kinetic energy. λ_t_ is the tangential acceleration eigenvalue: near obstacles, λ_t_ > 1, generating a tangential traction force to assist the robot in swiftly navigating past hazardous areas. The final generated vortex guidance vector is given by:

(22) $$v_{vortex}=M(p)\cdot v_{cmd}.$$

### 3.2. Multi-Dimensional Construction of an Enhanced Environmental Entrapment Index

To address the issue of global deadlock or limit cycle oscillations caused by a robot trapped in a deep U-shaped obstacle under significant time delays, a hybrid control architecture based on an environmental entrapment index is proposed in this study. This architecture autonomously seeks the direction of maximum free-space flux to guide the robot escape when a deadlock state is detected.

#### 3.2.1. Fusion Geometric Siege

To quantify the degree of geometric enclosure of the current environment, the environmental entrapment index Ω is defined. This index is calculated by evaluating the obstacle density within a local perception window:

(23) $$\Omega =\frac{1}{N}\sum_{i=1}^{N}\sigma (\frac{R_{max}-d_{i}}{R_{max}}),$$

where *N* is the number of LiDAR sampling points, *d_i_* is the range measurement in the *i-th* direction, *R_max_* is the effective detection radius, and σ(⋅) is a normalized weighting function. When the robot is deeply trapped, Ω→1; in open areas, Ω→0.

#### 3.2.2. Physical Stagnation

To quantify the robot actual motion response capability, the kinematic efficiency $\eta_{motion}$ within a time window $T_{w}$ is defined:

(24) $$\eta_{motion}(t)=\frac{\left\|P(t)-P(t-T_{w})\right\|}{\int_{t-T_{w}}^{t}\|v_{\mathrm{cmd}}(\tau )\|d\tau +\epsilon },$$

where **P**(*t*) is the robot’s position, and *v*_cmd_ is the operator command. When the operator provides continuous input but the robot displacement is minimal, the system is considered physically stuck. The stagnation index is defined as:

(25) $$\lambda_{stagnation}=e^{-k_{s}\cdot \eta_{motion}(t)}$$

As η_motion_ → 0, λ_stagnation_ → 1, indicating a high degree of stagnation.

#### 3.2.3. Interaction Conflict

Traditional measures of interaction coherence are often based on the cosine of the angle between two vectors:

(26) $$\cos\theta =\frac{v_{cmd}\cdot v_{out}}{\left\|v_{cmd}\|\|v_{out}\right\|}.$$

However, in deep U-shaped traps or when directly facing an obstacle, the robot’s actual velocity often approaches zero ($\|v_{out}\|\to 0$). In this case, cos *θ* becomes numerically singular, which can easily induce system chattering. To resolve this singularity issue, a compliance operator x based on inner product projection is introduced in this study. It is defined as the projection ratio of the actual motion vector onto the direction of the user command vector:

(27) $$x=\frac{v_{cmd}\cdot v_{out}}{\|v_{cmd}{\|}^{2}}.$$

The advantage of this definition lies in its denominator, which depends only on the user command $\left\|v_{cmd}\right\|$. As long as the operator provides input ($\|v_{cmd}\|\neq 0$), even if the robot is completely stuck ($\|v_{out}\|=0$), the operator *x* smoothly converges to 0, thereby eliminating the numerical singularity.

To transform the linear compliance *x* into a nonlinear conflict index, an adaptive mapping algorithm is designed. The input is saturated using a clamping function to confine it within the effective interval [0,1], filtering out interference from reverse motion or overshoot:

(28) $$x_{in}=\mathrm{clamp}(x,0,1)=\left\{\begin{array}{ll} 0, & x<0\\ x, & 0\leq x\leq 1\\ 1, & x>1 \end{array}\right..$$

To dynamically adjust sensitivity, an adaptive variable exponent γ dependent on the input state is constructed:

(29) $$\gamma =1+\sin\left(\frac{\pi }{2}\cdot x_{in}\right).$$

The final interaction conflict index *λ_conflict_* is generated using a modified cosine function:

(30) $$\lambda_{conf}=\frac{1+\cos(\pi \cdot x_{in}^{\gamma })}{2}.$$

This mapping curve exhibits excellent boundary characteristics. When fully compliant (*x*_in_ = 1, *γ* = 2, cos(*π*) = −1), *λ_conflict_* = 0, indicating no conflict. When completely stuck (*x*_in_ = 0, *γ* = 1, cos(*0*) = 1), *λ_conflict_* = 1, signifying severe conflict.

### 3.3. Composite Deadlock Criterion and Escape Activation

To ensure the robustness of the escape strategy activation, a composite deadlock index *D*_lock_(t) is constructed, integrating the three dimensions of geometric entrapment (Ω), physical stagnation (*λ_stagnation_*), and interaction conflict (*λ_conflictlict_*):

(31) $$D_{lock}(t)=\underset{\mathrm{Geometric}}{\underset{︸}{\Omega (t)}}\cdot \underset{\mathrm{Kinematic}}{\underset{︸}{\lambda_{stagnation}(t)}}\cdot \underset{\mathrm{Interaction}}{\underset{︸}{\lambda_{conflict}(t)}}.$$

A temporal confirmation mechanism is introduced into the system. Escape mode is triggered only when the composite index persistently exceeds a threshold:

(32) $$S_{escape}(t)=\left\{\begin{array}{l} 1,\;\;\;\;\mathrm{if}\;\int_{t-T_{trig}}^{t}I(D_{lock}(\tau )>D_{th})d\tau >T_{trig}\\ 0,\;\;\;\;\mathrm{otherwise} \end{array}\right.,$$

where I(⋅) is the indicator function, and *T_trig_* is the time threshold. This criterion dictates that the system is only determined to be in a deep trap when the environment is narrow (high Ω), the robot is physically immobilized (high *λ_stagnation_*), and human–robot intent is in severe conflict (high *λ_conflictlict_*) simultaneously. This effectively prevents frequent mode switching caused by fluctuations in a single metric during routine obstacle avoidance. The deadlock parameters are numerically defined as $D_{th}=0.5$ and $T_{trig}=1.5$ s, which were determined through extensive simulation experiments and comparative tuning. When the composite deadlock index *D*_lock_ continuously exceeds the threshold for the duration *T_trig_*, the system determines that a deep trap has been entered and that the IM-AVM local obstacle avoidance is ineffective. The system then switches to escape mode, employing a convolution-based distance flux algorithm to search for the locally optimal exit.

#### 3.3.1. Distance Potential Convolution and Maximum Flux Search

Unlike traditional binary gap search methods, a distance convolution algorithm that preserves depth information is adopted in this study, prioritizing escape channels that are both wide and deep. The free flux score $F_{k}$ for each direction is calculated by convolving the range sequence with a unit impulse response sequence $1_{W}$:

(33) $$F_{k}=(\hat{R}*1_{W})[k]=\sum_{j=-W/2}^{W/2}{\hat{r}}_{k+j},$$

where $R=\{r_{1},r_{2},\ldots ,r_{N}\}$ is the radar range data sequence, and ${\hat{r}}_{i}=\mathrm{clip}(r_{i},0,R_{view})$ with $R_{view}$ being the effective field-of-view depth. A larger $F_{k}$ value indicates not only the absence of obstacles in that direction but also a sufficiently wide angular opening in the open area. This effectively prevents the robot from mistakenly entering unsafe gaps. The index $k_{max}=\arg\max(F_{k})$ with the highest flux score is selected, and the locally optimal escape heading $\theta_{esc}$ is calculated:

(34) $$\theta_{esc}=\theta_{min}+k_{max}\cdot \Delta \theta_{res}+\theta_{robot}.$$

The corresponding unit escape vector is:

(35) $$u_{esc}={\left[\cos\theta_{esc},\sin\theta_{esc}\right]}^{T}.$$

Note that while this local heuristic ensures immediate reactive evasion, relying solely on it may lead to local minima in nested topologies; however, our proposed shared teleoperation framework inherently mitigates this limitation by leveraging the human operator’s global cognitive guidance.

#### 3.3.2. Dynamic Enhancement and State Reset

To overcome the nonlinear resistance from ground friction and motor dead zones in a deadlock state, the escape controller adopts the following guidance strategy: (Content truncated in provided image)

(36) $$v_{esc}=\eta_{boost}\cdot v_{base}\cdot u_{esc}.$$

Here $\eta_{boost}$ is the torque enhancement factor, which ensures that the robot possesses sufficient kinetic energy to overcome static friction at the moment of escape. Furthermore, to prevent overshoot caused by the accumulated sliding mode integral error during the deadlock phase, the controller’s integral state is reset at the instant the escape mode is activated:

(37) ∫e(τ)dτ←0,    if Mode→ESCAPE.

This mechanism eliminates the ejection effect and guarantees the smoothness of the escape process.

### 3.4. Dynamic Arbitration and Hierarchical Execution Architecture

To resolve authority conflicts between human operator intervention and autonomous machine control, a hierarchical arbitration framework is designed in this study. Based on the composite deadlock index D_lock_ and its duration, the system maintains two mutually exclusive control states:

Escape Takeover State: Upon deadlock confirmation, the machine assumes full control authority. The modulation weight is forcibly set to α = 1, and operator input is masked until the robot has completely exited the trap region (i.e., D_lock_ returns to a normal level).

Assisted Deadlock State: In non-deadlock conditions, the operator’s control authority is smoothly modulated using a continuous weight α. This weight is synthesized from a weighted combination of multi-dimensional risk factors:

(38) $$\alpha =S_{mooth}\left(w_{1}\cdot \Omega +w_{2}\cdot \lambda_{stagnation}+w_{3}\cdot \lambda_{conflict}\right),$$

where *S_mooth_* represents a first-order low-pass filter used to smooth weight fluctuations induced by perceptual noise. Based on the current state, the final reference velocity **v***_d_* output to the underlying sliding mode controller is defined as:

(39) $$v_{L}=\left\{\begin{array}{ll} (1-k_{\alpha }\alpha )\cdot v_{vortex}, & \mathrm{if}\;\mathrm{Mode}=\mathrm{ASSIST}\\ v_{\mathrm{esc}}, & \mathrm{if}\;\mathrm{Mode}=\mathrm{ESCAPE} \end{array}\right.\;.$$

### 3.5. Adaptive Hybrid Force Feedback Interaction Mechanism

Conventional artificial potential field methods, which directly map environmental repulsive forces to the haptic device, exhibit significant deficiencies: as the robot approaches an obstacle, the repulsive field increases sharply, causing severe oscillations in the haptic device; furthermore, within local minima regions, the feedback force directly opposes the operator’s intent. To address these issues, a hybrid force feedback dynamic adjustment algorithm is proposed in this study.

To ensure geometric consistency between the direction of haptic feedback and the operator first-person perspective, the global environmental force must be transformed into the robot’s body-fixed frame. The force vector transformation is defined as:

(40) $$F_{haptic}=R_{G}^{B}(\theta )\cdot F_{global}=\left[\begin{array}{cc} \cos\theta & \sin\theta \\ -\sin\theta & \cos\theta \end{array}\right]F_{global},$$

where $F_{global}$ and $F_{haptic}\in {\mathbb{R}}^{2}$ are the force vectors expressed in the global inertial frame and the local body-fixed frame, respectively; *θ* is the robot heading angle; and $R_{G}^{B}$ is the rotation matrix from the global frame to the body frame.

#### 3.5.1. Force Feedback in Assisted Deadlock State

In the assisted obstacle avoidance mode, the primary objective of force feedback is to accurately convey environmental information to the operator. Traditional methods exhibit stiffness singularity when approaching obstacles, where abrupt and intense force changes can easily destabilize system operation. Therefore, based on Equation (15), the avoidance force $F_{avoid}$ is defined as:

(41) $$F_{avoid}=K_{h}\cdot \ln(1+\|F_{env}\|)\cdot n_{env},$$

where **F***_env_* is the raw repulsive force based on APF, **n***_env_* is the unit direction vector, and *K_h_* is the force feedback gain coefficient. This model achieves robust nonlinear scaling of force perception: far from obstacles, sensor noise is effectively suppressed; during close-range interaction, the model provides a smooth and bounded damping sensation, fundamentally avoiding actuator saturation and high-frequency oscillations caused by stiffness singularity.

#### 3.5.2. Force Guidance in Escape Takeover State

When the deadlock detection algorithm is triggered, the system enters the escape takeover state. In this mode, to eliminate the interference of traditional repulsive forces with the operator’s escape maneuvers, the repulsive force channel is blocked, and a guidance force is generated instead. Based on Equation (36), the guidance force **F***_guide_* is defined as:

(42) $$F_{guide}=k_{guide}\cdot \frac{v_{esc}}{\left\|v_{esc}\right\|}\;,$$

where $k_{guide}$ is the guidance stiffness coefficient, and $v_{esc}/\left\|v_{esc}\right\|$ represents the optimal escape direction. This design provides the operator with only a subtle traction sensation in the escape direction, ensuring that the operator retains ultimate control authority throughout the escape process.

To mitigate high-frequency force jitter caused by random noise, a discrete-time first-order low-pass filter is introduced at the force output stage:

(43) $$F_{out}(k)=(1-\lambda )F_{out}(k-1)+\lambda F_{target}(k)\;.$$

The final output force is passed through an amplitude saturation stage to protect the hardware:

(44) $$F_{final}=\mathrm{sat}(F_{out},F_{max}).$$

#### 3.5.3. Predictive Damping Injection for Interaction

To address the passivity violation in the force feedback channel caused by reverse time delays, an adaptive damping injection based on the Smith predictor principle is introduced at the master side. The core of this mechanism is an internal prediction model $\hat{G}(s)$ that generates a zero-delay estimated force $F_{env}^{*}$ to compensate for the phase lag $T_{2}$ defined in Equation (12).

The internal model is constructed by linearizing the master-side impedance (Equation (10)) and integrating the state-dependent mapping logic. The transfer function of the predictor is explicitly stated as:

(45) $$\hat{G}(s)=\frac{F_{env}^{*}(s)}{f_{h}(s)}=\frac{K}{M_{m}s+B_{m}+K}$$

where $M_{m}$ and $B_{m}$ are the master physical parameters identified offline from Equation (10), and $f_{h}$ is the operator’s input. Crucially, the internal gain K is dynamically assigned to maintain consistency with the control mode: it is set to the haptic mapping equivalent of $F_{avoid}$ (Equation (41)) during assisted deadlock, and switches to the guidance logic $F_{guide}$ (Equation (42)) upon deadlock escape.

Based on the predicted force $F_{env}^{*}$, the master-side output force $f_{m}$ is designed as:

(46) $$f_{m}=F_{env}^{*}-B_{inj}(T_{RTT})\cdot {\dot{x}}_{m}.$$

The adaptive damping matrix **B***_inj_* is defined as:

(47) $$B_{inj}=b_{base}(1+\gamma T_{RTT})\cdot I_{2\times 2},$$

where **B***_inj_* represents the system energy dissipation port.

The synchronized switching of the internal model gain (Equations (41) and (42)) ensures that the estimated force $F_{env}^{*}$ remains task-consistent. As the round-trip time delay $T_{RTT}$ increases, the adaptive damping term $B_{inj}$ proactively dissipates the non-passive energy components. This ensures the closed-loop system satisfies the Passivity Criterion, fundamentally preventing oscillations and guaranteeing high-fidelity force reflection during complex remote maneuvers.

### 3.6. Follower Cooperative Control Based on Flow Field Modulation

The follower task is to maintain a specified geometric configuration relative to the leader within complex, unstructured environments. A cascaded control architecture comprising kinematic planning and dynamic tracking is designed in this section.

#### 3.6.1. Perception-Based Dynamic Formation Scaling

To achieve this adaptation, the scaling factor σ(t) is dynamically computed based on the local passage width $w_{obs}(t)$ perceived by the leader’s LiDAR:

(48) $$w_{obs}(t)=\min(R_{left}(t))+\min(R_{right}(t))$$

where $R_{left}$ and $R_{right}$ are the range measurements from the lateral sensing sectors. The raw scaling factor $\sigma_{raw}(t)$ is mapped from the detected width as follows:

(49) $$\sigma_{raw}(t)=\mathrm{sat}\left(\frac{w_{obs}(t)-d_{off}}{w_{nom}}\right)$$

where $d_{off}$ is the safety offset to account for the robot’s physical width, $w_{nom}$ is the nominal formation width, and the saturation function sat(⋅) constrains the output to $\left[\sigma_{min},1\right]$. To prevent formation collapse, a lower bound $\sigma_{min}$ is strictly enforced. To ensure geometric continuity, σ(t) is smoothed via:

(50) $$\sigma (t)=(1-\beta )\sigma (t-\Delta t)+\beta \sigma_{raw}(t)$$

where β is the smoothing factor. In the present study, the parameters are calibrated to match the simulated robot models within the Gazebo environment. Specifically, $d_{off}=0.2$ m and $w_{nom}=1.6$ m are determined by the virtual chassis radius and the nominal formation width, respectively. The lower bound $\sigma_{min}=0.35$ is set to prevent formation collapse, while the smoothing factor β=0.3 is empirically selected to balance reactive sensitivity with noise suppression.

#### 3.6.2. Anisotropic Flow Field Modulation

To address the potential conflict between formation maintenance and obstacle avoidance safety, an anisotropic flow field modulation strategy is proposed. The core concept is to dynamically modulate the raw formation command using the geometric characteristics of the environment. Initially, the nominal reference velocity is defined using Equation (16) and the gain $k_{p}$ from Equation (18). To prevent collisions in dense obstacles, an adaptive compliance mechanism relaxes $k_{p}$ based on the local repulsive field $R_{i}$ (Equation (15)), yielding the compliant raw velocity $v_{raw,i}$

(51) $$k_{p,i}^{*}=\frac{k_{p}}{1+\lambda_{c}\|R_{i}\|}$$

(52) $$v_{raw,i}={\dot{p}}_{d,i}+k_{p,i}^{*}e_{p,i}$$

where $\lambda_{c}>0$ is the compliance coefficient ($\lambda_{c}=2.0$). Substituting this compliant raw command $v_{raw,i}$ into Equation (21) for correction yields a safe modulated flow field $v_{safe,i}$:

(53) $$v_{safe,i}=M(q_{i})v_{raw,i}=\lambda_{n,i}(n_{i}^{T}v_{raw,i})n_{i}+\lambda_{t,i}(t_{i}^{T}v_{raw,i})t_{i}.$$

Equation (53) indicates that **M**(**q*_i_***) essentially functions as an anisotropic geometric filter. Based on the distribution of obstacles, it selectively attenuates the normal component of the raw command $v_{safe,i}$ that could lead to a collision, while preserving or enhancing the tangential component along the obstacle surface. When the formation traverses narrow passages or approaches obstacles, this modulation mechanism causes the actual velocity vector of the follower to deviate from the theoretical formation value. This implies that the system tolerates temporary geometric distortion of the formation as a trade-off to ensure obstacle avoidance safety.

This mechanism endows the multi-robot formation with a soft compression property. When encountering spatial constraints, robots temporarily slide away from their nominal positions to adapt to the environmental topology. Once the obstacle region is cleared ($\lambda_{n}\to 1$), the accumulated formation error is rapidly eliminated by the ANFTSMC, enabling automatic formation recovery.

#### 3.6.3. Adaptive Nonsingular Fast Terminal Sliding Mode Controller (ANFTSMC)

The modulated flow field $v_{safe,i}$ often exhibits non-smooth characteristics near obstacle boundaries. Furthermore, the Mecanum-wheeled chassis faces lumped uncertainties in practice, such as variations in ground friction and load perturbations. To achieve high-precision tracking of $v_{safe,i}$, a robust controller with finite-time convergence characteristics is designed. The velocity tracking error is defined as:

(54) $$e_{v,i}=v_{i}-v_{safe,i}.$$

Since directly differentiating the noisy signal $v_{safe,i}$ would amplify noise, a second-order fastest tracking differentiator (TD) is introduced to obtain a smooth acceleration estimate ${\dot{v}}_{safe,i}$. Taking the time derivative of Equation (53) and substituting Equation (6), the open-loop system error dynamics are obtained after rearrangement:

(55) $${\dot{e}}_{vi}=f(v_{i})+Bu_{i}+d_{i}-{\dot{v}}_{safe,i}\;.$$

To avoid the singularity of traditional terminal sliding mode and to enhance convergence speed, an integral-type nonsingular fast terminal sliding surface **s***_i_* is selected:

(56) $$s_{i}=e_{vi}+\int_{0}^{t}\left(\alpha e_{vi}+\beta |e_{vi}{|}^{\gamma }\mathrm{sgn}(e_{vi})\right)d\tau \;,$$

where $\mathrm{sig}{\left(x\right)}^{\gamma }=|x{|}^{\gamma }\mathrm{sgn}(x)$. The linear term $\alpha e_{vi}$ dominates the dynamics far from the equilibrium point, ensuring rapid exponential convergence of the error towards the sliding surface. The nonlinear term $\beta |e_{vi}{|}^{\gamma }\mathrm{sgn}(e_{vi})$ (with 1<p/q<2) dominates the dynamics near the equilibrium point, ensuring precise convergence of the error to zero within finite time.

The final control torque is designed as $\tau_{i}=\tau_{eq,i}+\tau_{rob,i}$. The equivalent control term $\tau_{eq,i}$ compensates for the nominal model dynamics:

(57) $$\tau_{eq,i}=C_{i}v_{i}+D_{i}v_{i}+M_{i}\left({\dot{v}}_{safe,i}+c_{1}e_{vi}+c_{2}\mathrm{sig}{\left(e_{vi}\right)}^{p/q}\right).$$

The robust control term $\tau_{rob,i}$ is designed to suppress lumped disturbances. Considering that the high-frequency switching of the sign function sgn(⋅) in traditional sliding mode control can induce chattering, leading to actuator wear and controller signal oscillations, a saturation function sat(⋅) is employed to replace the sign function, and an adaptive gain ${\hat{\delta }}_{i}$ is introduced to estimate the disturbance upper bound:

(58) $$\tau_{rob,i}=-({\hat{\delta }}_{i}+\eta )\cdot \mathrm{sat}(s_{i},\Phi ),\;\;\;\;\mathrm{sat}(s_{i},\Phi )=\left\{\begin{array}{ll} \frac{s_{i}}{\Phi }, & \|s_{i}\|<\Phi \\ \frac{s_{i}}{\left\|s_{i}\right\|}, & \|s_{i}\|\geq \Phi \end{array}\right..$$

To cope with unknown disturbance bounds, the adaptive law is designed as ${\dot{\hat{\delta }}}_{i}=\mu \|s_{i}\|$, where *μ* > 0 is the adaptive rate parameter. The saturation function provides a continuous linear transition of the control effort within the boundary layer Φ.

Combining Equations (57) and (58), the final control torque input $\tau_{i}$ for the *i*-th follower is obtained:

(59) $$\tau_{i}=C_{i}v_{i}+D_{i}v_{i}+M_{i}\left({\dot{v}}_{safe,i}+c_{1}e_{vi}+c_{2}\mathrm{sig}{\left(e_{vi}\right)}^{p/q}\right)-({\hat{\delta }}_{i}+\eta )\mathrm{sat}(s_{i},\Phi ).$$

Equation (59) manifests as a flexible switching of motor torque, significantly mitigating high-frequency chattering in the control signal while preserving robustness. Concurrently, the adaptive law ensures that the gain is dynamically adjusted according to the current tracking error, circumventing the conservatism inherent in traditional fixed-gain approaches.

Consequently, this section establishes a human–robot-environment symbiotic control strategy that addresses the dual challenges posed by time delays and unstructured environments. For the leader subsystem, an intent-mediated asymmetric dynamic shared control is proposed. Local saddle point deadlocks are resolved via the topological chirality factor σ*_chirality_*, while global U-shaped trap issues are addressed through the composite deadlock criterion, enabling mode switching between assisted deadlock and escape takeover. For the follower subsystem, a cooperative control based on flow field modulation is established. The dynamic modulation matrix resolves the coupling conflict between formation maintenance and obstacle avoidance. The ANFTSMC, combined with a tracking differentiator (TD) and a saturation function, guarantees high-precision, chatter-free tracking under model uncertainties, achieving an organic unification of safety, robustness, and transparency.

## 4. System Stability and Convergence Analysis

This section aims to provide a rigorous theoretical analysis of the shared control and formation obstacle avoidance strategy for teleoperation proposed in Section 3. The proof follows a three-tiered logic: first, based on energy passivity theory, the interaction stability of the leader under communication delays and damping injection is demonstrated; second, based on the invariant set principle, the obstacle avoidance safety of the followers under flow field modulation is proven; finally, using finite-time Lyapunov stability theory, the convergence of the follower dynamic system to the safe trajectory and its robustness against disturbances are established.

For the subsequent proofs, the following core lemma is introduced:

**Lemma 1** (Finite-Time Stability). *Consider the nonlinear system* $\dot{x}=f(x)$. *If there exists a continuous positive definite function V(x) and constants* c>0,0<α<1 *such that the differential inequality holds:*

(60) $$\dot{V}(x)+cV{\left(x\right)}^{\alpha }\leq 0,$$

*then the system state converges to the origin within a finite time* $T_{r}$*, and the upper bound of the convergence time is given by:*

(61) $$Tr\;\leq \frac{V{\left(x_{0}\right)}^{1-\alpha }}{c(1-\alpha )}.$$

### 4.1. Passivity Proof of Leader Interaction

For the Smith predictor-based damping injection strategy proposed in Section 3.1, it must be proven that the master-side closed-loop system remains passive from the perspective of the operator, even in the presence of communication delays ($T_{delay}>0$). That is, the system does not actively generate energy due to the phase lag introduced by the delay, thereby ensuring the stability of human–robot interaction.

**Theorem 1.**  *Consider the master-side dynamic system Equation (9) and the damping injection control law Equations (45) and (46). If the adaptive damping matrix* $B_{inj}$ *is designed to satisfy the dissipation condition* $P_{diss}\geq P_{active}$ *, then the mapping from the human input force* $f_{h}$ *to the haptic device velocity* ${\dot{x}}_{m}$ *satisfies the passivity inequality:*

(62) $$\int_{0}^{t}f_{h}^{T}{\dot{x}}_{m}d\tau \geq -V.$$

**Proof.**  To quantitatively analyze the energy flow characteristics of the system, an energy storage function is first constructed. The generalized kinetic energy of the master system is selected as a Lyapunov candidate function *V*(**x***_m_*). Utilizing the positive definiteness of the inertia matrix **M***_m_*, it can be defined as:

(63) $$V(x_{m})=\frac{1}{2}{\dot{x}}_{m}^{T}M_{m}{\dot{x}}_{m}.$$

□

Taking the time derivative of this energy function and introducing the closed-loop dynamic constraints. The master-side dynamic Equation (9) is substituted into the expression for ˙*V*˙. Furthermore, considering Equation (46), where the control torque **f***_m_* consists of the Smith-predicted environmental force $F_{env}^{*}$ and the actively injected damping $-B_{inj}{\dot{x}}_{m}$. Expanding **f***_m_* and substituting, the power flow equation of the system is obtained:

(64) $$\dot{V}={\dot{x}}_{m}^{T}f_{h}+{\dot{x}}_{m}^{T}(F_{env}^{*}-B_{inj}{\dot{x}}_{m}-B_{m}{\dot{x}}_{m}).$$

To clarify the impact of delay on stability, the terms in Equation (64) are reorganized according to their physical significance, yielding three key power terms: the external input power $P_{in}={\dot{x}}_{m}^{T}f_{h}$, the total dissipated power:

(65) $$P_{diss}={\dot{x}}_{m}^{T}(B_{m}+B_{inj}){\dot{x}}_{m}.$$

Therefore, the spurious active power introduced by the delayed environmental force is expressed as $P_{active}={\dot{x}}_{m}^{T}F_{env}^{*}$. Accordingly, the power equation is rewritten as:

(66) $$\dot{V}=P_{in}-P_{diss}+P_{active}.$$

Based on this, a dissipation-dominant condition is introduced. In the presence of communication delays, phase lag may lead to $P_{active}>0$, i.e., the system outputs energy, causing oscillations. However, the adaptive law ensures that the damping matrix $B_{inj}$ increases monotonically with the round-trip delay $T_{rtt}$, thereby guaranteeing that the total dissipated power always covers the spurious active power:

(67) $$P_{diss}\geq P_{active}\Rightarrow {\dot{x}}_{m}^{T}(B_{m}+B_{inj}){\dot{x}}_{m}\geq {\dot{x}}_{m}^{T}F_{env}^{*}.$$

Finally, the passivity conclusion is derived using integral inequalities. Substituting condition Equation (67) into Equation (66) yields the inequality:$\dot{V}\leq P_{in}$. Integrating this over the time interval [0, t]:

(68) $$V(t)-V(0)\leq \int_{0}^{t}f_{h}^{T}{\dot{x}}_{m}d\tau .$$

Since kinetic energy V(t) ≥ 0, the above implies that the energy absorbed by the system from the external environment is always greater than or equal to the increment in the system’s stored energy. This demonstrates that the closed-loop system does not actively generate energy under delayed conditions and strictly satisfies the definition of passivity.

### 4.2. Proof of Forward Invariance for Obstacle Avoidance Safety

In Section 3, an anisotropic flow field modulation strategy was employed for the followers to ensure that the robot always navigates within the safe set $S_{free}=\{q|D(q)>0\}$. The forward invariance of this safe set is now proven using the Nagumo set invariance theorem.

**Theorem 2.**  *The free space outside obstacles is defined as the open set* $S_{free}=\{q|D(q)>0\}$ *, with its boundary given by* ∂S={q∣D(q)=0} *. If the motion of a robot* k∈{L,1,…,n} *(where L denotes the leader and I denotes the i-th follower) is governed by the anisotropic modulation matrix* **M**(**q***_k_) such that* ${\dot{q}}_{k}=M(q_{k})v_{in,k}$ *, with *$v_{in,k}$ *being an arbitrary bounded input command—specifically, for the leader,* $v_{in,L}=v_{cmd}$ *, and for a follower,* $v_{in,i}=v_{raw,i}$ *—then* $S_{free}$ *is a forward invariant set for the system.*

**Proof.**  According to Nagumo theorem, the necessary and sufficient condition for the set $S_{free}$ to remain forward invariant is that when the system state is on the boundary $q_{k}\in \partial S$, its velocity vector ${\dot{q}}_{k}$ must point into the tangent space $T_{\partial S}\left(q_{k}\right)$ or into the interior of the free space. That is, it must be shown that:

(69) $$\dot{D}(q_{k})=\nabla D{\left(q_{k}\right)}^{T}{\dot{q}}_{k}\geq 0,\;\;\;\;\forall q_{k}\in \partial S.$$

□

It is noted that the gradient of the distance field $\nabla D(q_{k})$ is precisely the unit outward normal vector $n_{k}$ on the obstacle surface. Substituting the control law ${\dot{q}}_{k}=M(q_{k})v_{in,k}$ into Equation (63) yields:

(70) $$\dot{D}(q_{k})=n_{k}^{T}M(q_{k})v_{in,k}..$$

Utilizing the spectral decomposition form of $M(q_{k})$ from Section 3:

(71) $$M(q_{k})=\lambda_{n,k}n_{k}n_{k}^{T}+\lambda_{t,k}t_{k}t_{k}^{T},$$

expanding and substituting into the above equation gives:

(72) $$\dot{D}(q_{k})=n_{k}^{T}\left(\lambda_{n,k}n_{k}n_{k}^{T}+\lambda_{t,k}t_{k}t_{k}^{T}\right)v_{in,k}\;.$$

Applying the distributive property of the vector inner product and the orthogonality of the basis vectors, the term containing the tangent vector **t***_k_* vanishes due to orthogonality:

(73) $$\begin{array}{l} \dot{D}(q_{k})=\lambda_{n,k}(n_{k}^{T}n_{k})(n_{k}^{T}v_{in,k})+\lambda_{t,k}(n_{k}^{T}t_{k})(t_{k}^{T}v_{in,k})\\ =\lambda_{n,k}\cdot 1\cdot (n_{k}^{T}v_{in,k})+\lambda_{t,k}\cdot 0\cdot (t_{k}^{T}v_{in,k})\\ =\lambda_{n,k}(n_{k}^{T}v_{in,k})\; \end{array}.$$

According to the eigenvalue design rule, as the robot approaches the obstacle boundary (i.e., $D(q_{k})\to 0$), the normal eigenvalue satisfies: $\underset{D\to 0}{\lim}\lambda_{n,k}=0$. Therefore, at the boundary:

(74) $${\left.\dot{D}(q_{k})\right|}_{q_{k}\in \partial S}=0\cdot (n_{k}^{T}v_{in,k})=0\;.$$

Conclusion: Equation (68) indicates that, regardless of the direction of the input command $v_{in,k}$, the normal component of the system’s velocity at the boundary is strictly nullified. This means the velocity vector ${\dot{q}}_{k}$ is always tangent to the obstacle boundary (${\dot{q}}_{k}\in T_{\partial S}$), satisfying Nagumo invariance condition. Consequently, any trajectory initialized within $S_{free}$ cannot cross the boundary ∂S.

### 4.3. Finite-Time Convergence Proof of Follower Dynamics

This subsection demonstrates that the proposed Adaptive Nonsingular Fast Terminal Sliding Mode Controller (ANFTSMC) guarantees that the system converges to the safe flow field $v_{safe,i}$ within a finite time.

**Theorem 3.**  *Consider the error dynamic system for follower i. Under the action of the control torque from Equations (57) and (58) and the adaptive law* ${\dot{\hat{\delta }}}_{i}=\mu \|s_{i}\|$ *, the sliding variable* $s_{i}$ *and the velocity error* ${\dot{e}}_{i}$ *are Uniformly Ultimately Bounded (UUB), converging to the boundary layer* $|s_{i}|\leq \Phi$ *in finite time.* 

**Proof.**  The proof is structured in two phases. □

1. Sliding Mode Reaching Phase

A Lyapunov candidate function is selected, incorporating the generalized kinetic energy of the *i*-th robot and the estimation error of the adaptive parameter:

(75) $$V_{s,i}=\frac{1}{2}s_{i}^{T}M_{i}s_{i}+\frac{1}{2\mu }{\tilde{\delta }}_{i}^{2},$$

where ${\tilde{\delta }}_{i}={\hat{\delta }}_{i}-\delta_{max}$ is the estimation error, and $\delta_{max}$ is the true upper bound constant of the disturbance $\tau_{d,i}$, satisfying $\|\tau_{d,i}\|\leq \delta_{\max}$.

Taking the time derivative, utilizing the skew-symmetric property of robot dynamics ($x^{T}({\dot{M}}_{i}-2C_{i})x=0$), and substituting the closed-loop dynamics yields:

(76) $${\dot{V}}_{s,i}=s_{i}^{T}M_{i}{\dot{s}}_{i}+\frac{1}{2}s_{i}^{T}{\dot{M}}_{i}s_{i}+\frac{1}{\mu }{\tilde{\delta }}_{i}{\dot{\hat{\delta }}}_{i}=s_{i}^{T}(\tau_{rob,i}-\tau_{d,i})+{\tilde{\delta }}_{i}\|s_{i}\|.$$

Note: This derivation assumes that the equivalent control $\tau_{eq,i}$ precisely cancels the nominal model dynamics **C*_i_***, **D*_i_*** and the reference terms. Substituting Equation (58) and considering the condition outside the boundary layer ($\|s_{i}\|\geq \Phi$) yields:

(77) $$\begin{array}{l} {\dot{V}}_{s,i}=s_{i}^{T}\left[-({\hat{\delta }}_{i}+\eta )\frac{s_{i}}{\left\|s_{i}\right\|}-\tau_{d,i}\right]+({\hat{\delta }}_{i}-\delta_{max})\|s_{i}\|\\ =-({\hat{\delta }}_{i}+\eta )\|s_{i}\|-s_{i}^{T}\tau_{d,i}+{\hat{\delta }}_{i}\|s_{i}\|-\delta_{max}\|s_{i}\|\\ \leq -\eta \|s_{i}\|-\delta_{max}\|s_{i}\|+\|s_{i}\|\|\tau_{d,i}\|\\ \leq -\eta \|s_{i}\|\; \end{array}.$$

According to the Rayleigh-Ritz theorem, there exists a constant $\lambda_{min}(M_{i})>0$ such that:

(78) $$\frac{1}{2}\lambda_{min}(M_{i})\|s_{i}{\|}^{2}\leq \frac{1}{2}s_{i}^{T}M_{i}s_{i}\leq V_{s,i},$$

which implies:

(79) $$\|s_{i}\|\geq \sqrt{\frac{2}{\lambda_{max}(M_{i})}}V_{s,i}^{1/2}.$$

Defining $\kappa =\eta \sqrt{\frac{2}{\lambda_{max}(M_{i})}}$, the following inequality is obtained:

(80) $${\dot{V}}_{s,i}+\kappa V_{s,i}^{1/2}\leq 0.$$

By Lemma 1, the system state reaches the boundary layer $|s_{i}|\leq \Phi$ in finite time $t_{r}$. Furthermore, replacing the signum function with $sat(s_{i},\Phi )$ yields practical Uniformly Ultimately Bounded stability. This theoretical relaxation is an intentional engineering trade-off to suppress high-frequency actuator chattering while preserving acceptable tracking precision.

2. Sliding Mode Phase

Once the system enters the sliding mode, it satisfies $s_{i}=0$ and ${\dot{s}}_{i}=0$. Differentiating the sliding surface definition Equation (56) with respect to time yields the error dynamics for follower *i*:

(81) $${\dot{e}}_{vi}=-c_{1}e_{vi}-c_{2}\mathrm{sig}{\left(e_{vi}\right)}^{p/q}.$$

A Lyapunov function for the error state is selected:

(82) $$V_{e,i}=\frac{1}{2}e_{vi}^{T}e_{vi}.$$

Taking its time derivative:

(83) $${\dot{V}}_{e,i}=e_{vi}^{T}{\dot{e}}_{vi}=-c_{1}e_{vi}^{T}e_{vi}-c_{2}e_{vi}^{T}\mathrm{sig}{\left(e_{vi}\right)}^{p/q}.$$

Noting that $e_{vi}^{T}\mathrm{sig}{\left(e_{vi}\right)}^{p/q}=\|e_{vi}{\|}^{1+p/q}={\left(2V_{e,i}\right)}^{\frac{p+q}{2q}}$, and since $c_{1}>0$, it follows that:

(84) $${\dot{V}}_{e,i}\leq -c_{2}2^{\frac{p+q}{2q}}V_{e,i}^{\frac{p+q}{2q}}.$$

Given the parameter design satisfies p < q, the exponent α = *p* + *q*/2*q* satisfies 0.5 < α < 1. This inequality perfectly conforms to the finite-time convergence condition of Lemma 1. Therefore, the velocity tracking error $e_{vi}$ of the *i*-th follower converges precisely to zero within a finite time, enabling accurate replication of the safe formation flow field.

In summary, it is first demonstrated that the adaptive damping injection mechanism effectively compensates for the non-passivity introduced by communication delays, ensuring the energy stability of the human–robot closed loop. Second, it is proven that the eigenvalue configuration within the IM-AVM strategy geometrically guarantees the forward invariance of the safe set, ensuring zero collisions for the robot. Finally, it is established that the ANFTSMC algorithm, combined with the adaptive law, guarantees finite-time convergence of the sliding variable and the tracking error.

## 5. Experimental Verification and Results Analysis

To validate the feasibility and robustness of the proposed shared control framework, a simulated verification platform was developed. This platform establishes a closed-loop teleoperation environment by integrating a physical haptic device with a virtual simulation engine. Specifically, the master side incorporates a physical Geomagic Touch haptic device, which captures the operator’s motion intent via 1 kHz high-frequency hardware sampling and renders virtual haptic feedback from the slave side to satisfy the transparency requirements of the teleoperation system. Meanwhile, the master and slave sides are deployed on independent computing nodes connected via an Ethernet router, utilizing the ROS 2 (DDS) middleware to achieve cross-machine asynchronous communication. On the slave side, the controlled multi-robot formation operates within the Gazebo 11 physics engine. Each robot model is constructed based on the kinematic parameters of the physical platform, responsible for processing complex formation evolution and obstacle avoidance logic, while feeding real-time states back to the master side to complete the closed-loop human–robot interaction control. The critical tunable parameters of the controllers, shared control arbitration, and IM-AVM are comprehensively summarized in Table 1 and Table 2.

### 5.1. Teleoperation Mapping Evaluation

This experiment aimed to verify the accuracy of the master-slave motion mapping in omnidirectional translation mode. The operator’s forward and backward manipulation of the haptic device was mapped to the longitudinal linear velocity *v_x_* of the robot, while lateral left-right movements were mapped to the lateral velocity *v_y_*.

**Figure 4:**
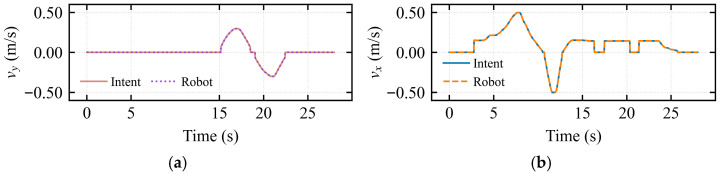
Velocity mapping in omnidirectional translation mode. (**a**) Longitudinal linear velocity. (**b**) Lateral velocity.

Figure 4a presents the longitudinal linear velocity response. The solid line represents the intended velocity input from the operator via the haptic device, and the dashed line represents the velocity commanded to the robot. When continuous positive and negative velocity commands were applied by the operator, a high degree of consistency was observed between the mapped curve and the intended trajectory.

Figure 4b displays the lateral velocity response. Analysis of the curves indicates that during the acceleration phase from 15 s to 18 s, as well as the deceleration and reverse phases from 19 s to 22 s, the intended signal and the mapped velocity nearly completely overlapped, with no perceptible delay. This minimal latency ensures transparency during teleoperation, enabling the operator to perceive the robot’s motion state in real time and thereby establish an effective closed-loop feedback control.

### 5.2. Core Algorithm Verification

**Figure 5:**
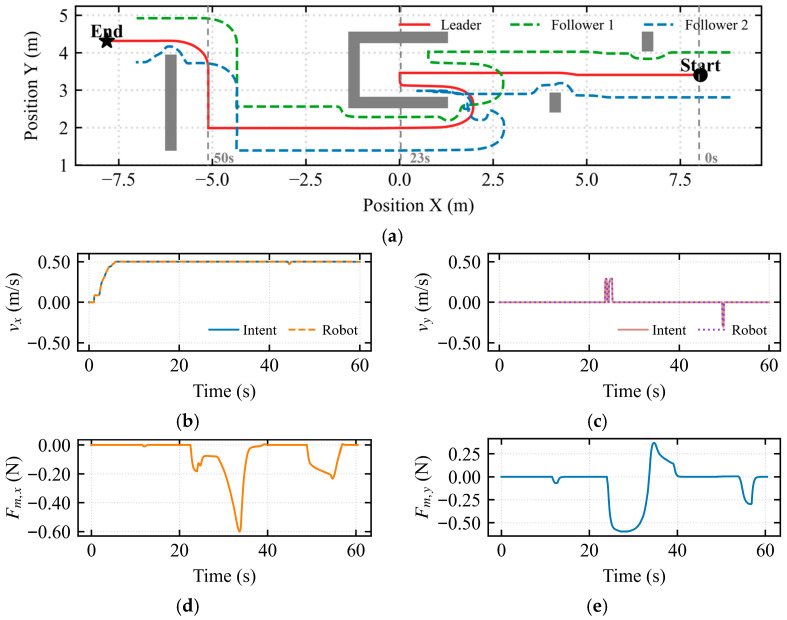
Assisted deadlock in the U-shaped trap scenario. (**a**) Assisted deadlock trajectory. (**b**) Longitudinal linear velocity. (**c**) Lateral velocity. (**d**) Longitudinal force. (**e**) Lateral force.

To address the inherent limitation of the traditional Artificial Potential Field (APF) method, which is prone to entrapment in local minima, a U-shaped trap scenario was constructed for testing, as depicted in Figure 5.

The trajectory results shown in Figure 5a demonstrate that the dynamic asymmetric modulation matrix effectively breaks the symmetry of the potential field, enabling successful obstacle avoidance at the mouth of the U-shaped trap. The followers adapted their formation through autonomous contraction, avoiding collisions with side walls while maintaining formation integrity. Figure 5b indicates that the mapped longitudinal velocity was consistently maintained at approximately 0.5 m/s, indicating continuous forward motion of the simulated vehicle throughout the assisted deadlock state. In Figure 5c, pulses of approximately 0.25 m/s and −0.25 m/s were observed near *t* ≈ 23 s and *t* ≈ 50 s, respectively. These pulses demonstrate that the intent-mediated asymmetric dynamic shared control leverages the operator’s micro-intentions to dynamically determine the flow field’s rotational direction, thereby breaking the potential field symmetry. Figure 5d quantifies the environmental constraint information perceived by the operator in the longitudinal dimension. During the interval from 25 s to 40 s, corresponding to the deepest entrapment of the robot within the U-shaped trap, a negative peak of −0.6 N was observed in the longitudinal force feedback. Figure 5e quantifies the environmental constraint information perceived in the lateral dimension. During the interval from 25 s to 33 s, corresponding to obstacle avoidance via the intent-mediated asymmetric dynamic shared control after the robot entered the local minimum, a negative peak of −0.6 N was observed in the lateral force feedback.

**Figure 6:**
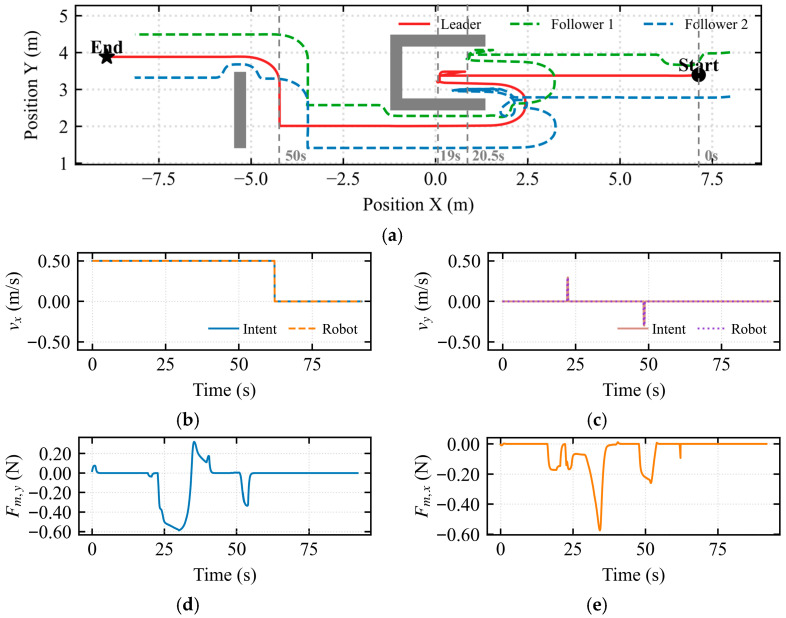
Escape takeover in the U-shaped trap scenario. (**a**) Escape takeover trajectory. (**b**) Longitudinal linear velocity. (**c**) Lateral velocity. (**d**) Longitudinal force. (**e**) Lateral force.

As shown in Figure 6a, during the escape takeover state, the operator intent was guided by the haptic force. The vehicle was determined to be trapped in the U-shaped obstacle at position 0.0 m. Upon entering the escape takeover state, the system searched for the locally optimal exit and executed a retreat of 0.5 m to capture the operator’s attention, ultimately leading to successful escape from the U-shaped trap. Therefore, the dynamic asymmetric modulation matrix is capable of breaking the potential field symmetry at the U-shaped mouth, searching for the locally optimal exit based on the composite deadlock criterion and escape activation mechanism. This also demonstrates that the proposed shared control framework successfully identified the non-convex geometric characteristics of the environment and generated a smooth escape trajectory tangent to the obstacle boundaries.

Figure 6b shows that the mapped longitudinal velocity remained consistently at 0.5 m/s before 60 s. In Figure 6c, pulses of approximately 0.25 m/s and −0.25 m/s were observed near *t* ≈ 22 s and *t* ≈ 50 s, respectively. This indicates that the intent-mediated asymmetric dynamic shared control utilizes the operator’s micro-intentions to dynamically determine the flow field’s rotational direction, thereby breaking the potential field symmetry. Figure 6d quantifies the environmental constraint information perceived by the operator in the longitudinal dimension. During the interval from 25 s to 42 s, corresponding to the deepest entrapment within the U-shaped trap, a negative peak of −0.6 N was observed in the longitudinal force feedback. Figure 6e quantifies the lateral environmental constraint information. During the interval from 23 s to 42 s, corresponding to the period when the escape takeover state alerted the operator and the intent-mediated asymmetric dynamic shared control was activated to break the potential field symmetry, a negative peak of −0.32 N was observed in the lateral force feedback.

To further investigate the interaction characteristics of the hierarchical shared control framework, two sets of experiments were designed: the standard obstacle avoidance mode (assisted deadlock) and the escape mode, as illustrated in the figures. In both sets of experiments, the robot formation successfully traversed the U-shaped trap region. However, the underlying deadlock resolution mechanisms and human–robot collaboration modes were fundamentally different.

**Figure 7:**
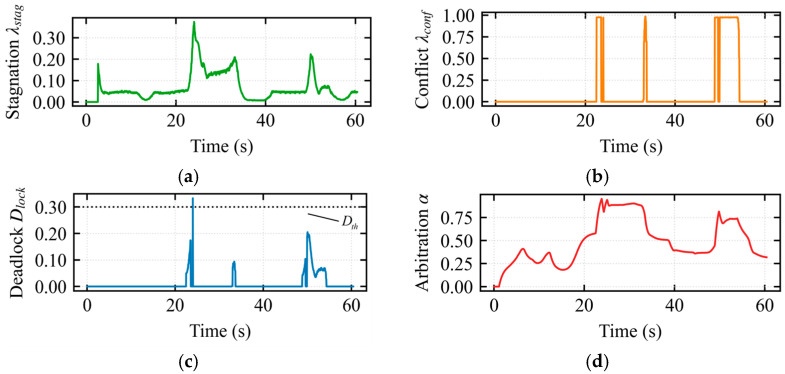
Conflict and arbitration mechanism in the assisted deadlock state. (**a**) Stagnation index *λ_stagnation_*. (**b**) Interaction conflict index *λ_conflict_.* (**c**) Deadlock index D*_lock_.* (**d**) Arbitration factor α.

In Figure 7a, the stagnation index varied continuously during the assisted deadlock state, reaching a peak of 0.38 around 23 s. At this moment, continuous input was provided by the operator while the robot’s displacement was minimal. In Figure 7b, during the intervals 22–25 s, 33–34 s, and 49–55 s, the interaction conflict index *λ_conflict_* = 1 was 1, indicating severe conflict. In Figure 7c, at 23 s, the deadlock index exceeded the threshold of 0.3. However, the escape mode was not triggered, as the composite index did not continuously exceed the threshold within the temporal confirmation mechanism. This demonstrates that the temporal confirmation mechanism effectively filters out noise, preventing false activations and enhancing the robustness of the composite deadlock criterion and escape mechanism. In Figure 7d, the arbitration factor *α* smoothly regulated the operator’s control authority during the assisted deadlock state. The arbitration factor exceeded 0.75 during the 22–33 s interval but never reached 1, confirming that the system remained in the assisted deadlock state.

**Figure 8:**
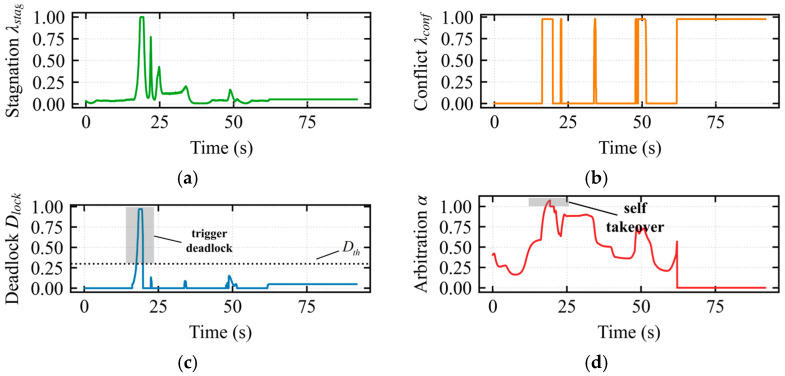
Conflict and arbitration mechanism in the escape takeover state. (**a**) Stagnation index *λ_stagnation_*. (**b**) Interaction conflict index *λ_conflict_*. (**c**) Deadlock index D*_lock_.* (**d**) Arbitration factor α.

In Figure 8a, the stagnation index reached its maximum around 19 s, corresponding to the largest discrepancy between operator input and robot displacement, indicating severe system stagnation. In Figure 8b, during the 19–20 s interval, the interaction conflict index *λ_conflict_* = 1 was 1, indicating severe conflict. In Figure 8c, at 19–20 s, the deadlock index reached 1, and the composite index continuously exceeded the threshold within the temporal confirmation mechanism, triggering the escape takeover state. In Figure 8d, during the 19–20 s interval, upon deadlock confirmation, the vehicle assumed full control authority with the modulation weight *α* = 1, and operator input was masked until the robot completely exited the trap region (i.e., $D_{lock}$ returned to normal).

In the escape takeover state, the operator was enabled to perceive the topological structure of the trap, thereby being intuitively guided to cooperate with the algorithm for successful escape.

**Figure 9:**
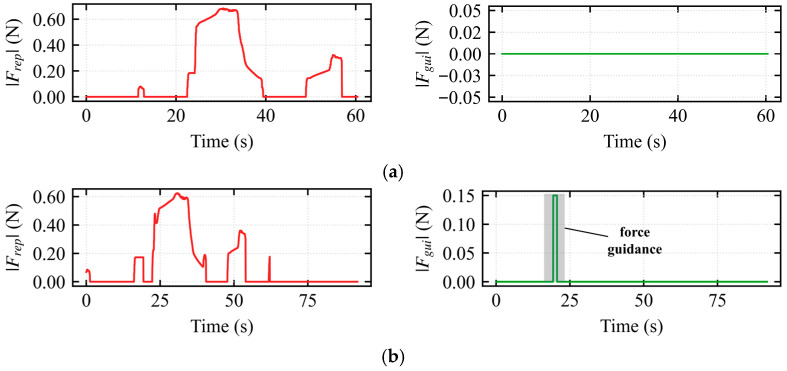
Force feedback in the U-shaped trap scenario. (**a**) Force feedback in the assisted deadlock state. (**b**) Force feedback in the escape takeover state.

In Figure 9a, with the vehicle in assisted obstacle avoidance mode, only the avoidance force *F_avoid_* was active, accurately conveying environmental risk information to the operator. This avoidance force reached a peak of 0.7 at 30 s, achieving robust nonlinear scaling for motion suppression and enabling smooth obstacle avoidance. In Figure 9b, the vehicle entered the escape takeover state during the 19–20 s interval. At this time, the avoidance force *F_avoid_* was zero, while the guidance force *F_guide_* was 0.15, providing the operator with a subtle *traction* sensation in the escape direction.

**Figure 10:**
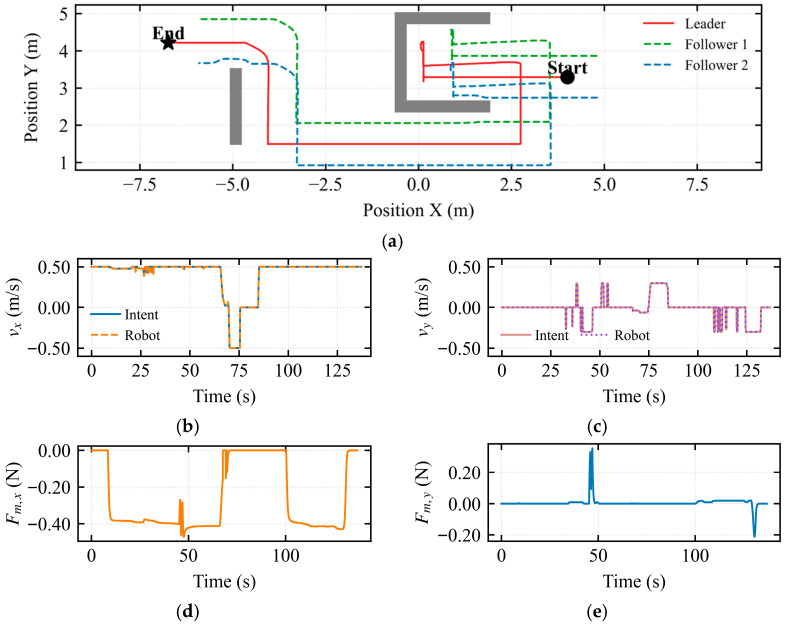
Conventional APF in the U-shaped trap scenario. (**a**) trajectory Conventional APF. (**b**) Longitudinal linear velocity. (**c**) Lateral velocity. (**d**) Longitudinal force. (**e**) Lateral force.

Figure 10a shows the trajectory under conventional APF, where the formation is trapped in local minima at the U-shaped obstacle and the long wall. Successful navigation in this baseline case relies entirely on manual teleoperative override. Figure 10b,c display the mapped longitudinal and lateral velocities, revealing that persistent operator inputs are required to escape the stagnation zones, leading to increased task duration. Figure 10d,e quantify the haptic force feedback rendered to the operator in the longitudinal and lateral dimensions, respectively. To further validate the robust navigation capabilities of the proposed framework, a comparative study was conducted between the traditional APF and the IM-AVM strategy within complex U-shaped traps and long-wall environments. Under traditional APF, the multi-robot formation frequently stagnated at local minima near the concave regions of the U-shaped obstacles. Escaping these traps required the operator to provide continuous and intensive corrective commands, leading to a significantly longer mission completion time compared to the IM-AVM approach. In contrast, the IM-AVM strategy effectively utilizes the operator’s transient micro-intention force as a symmetry-breaking mechanism, allowing the formation to autonomously navigate out of the deadlock with minimal human intervention. Consequently, the proposed IM-AVM-based shared control framework demonstrates superior efficiency and reduced operator dependency in resolving the local minima problem.

### 5.3. Compliant Formation Deformation and Trajectory Adaptation

**Figure 11:**
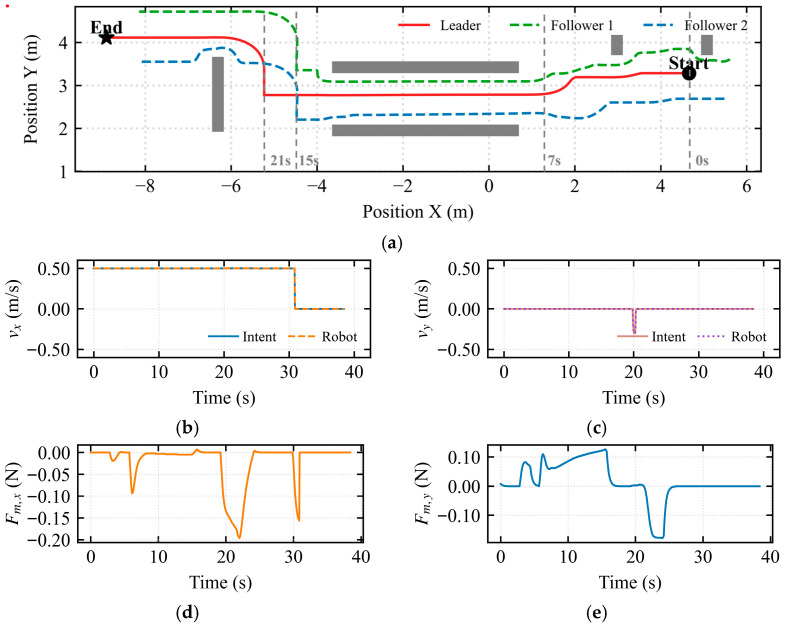
Formation transformation in the narrow corridor. (**a**) Narrow passage trajectory. (**b**) Longitudinal linear velocity. (**c**) Lateral velocity. (**d**) Longitudinal force. (**e**) Lateral force.

As shown in Figure 11, a test scenario was configured where the environment transitioned from a wide area to a narrow corridor.

**Figure 12:**
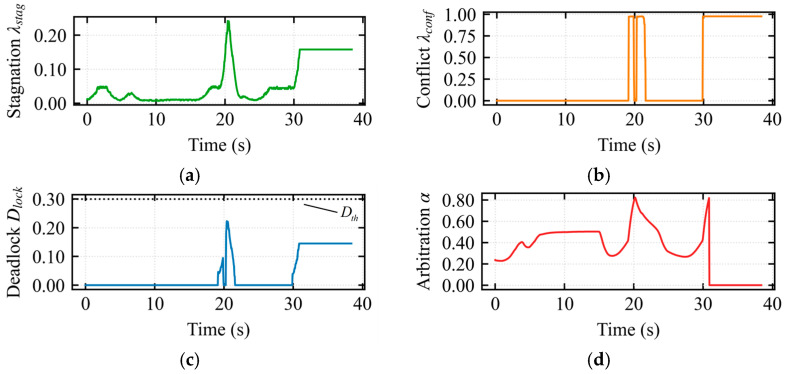
Response of core state variables to geometric constraints. (**a**) Stagnation index *λ_stagnation_.* (**b**) Interaction conflict index *λ_conflict_.* (**c**) Deadlock index D*_lock_.* (**d**) Arbitration factor α.

The formation trajectory in Figure 11a illustrates a complete compliant deformation process: formation-based obstacle avoidance, individual follower obstacle avoidance, formation maintenance during passage through the narrow corridor (X∈[−4,1]), and subsequent elastic recovery. Figure 11b indicates that the mapped longitudinal velocity was consistently maintained at approximately 0.5 m/s. In Figure 11c, a pulse of approximately 0.25 m/s was observed near t ≈ 21 s, resulting from a brief lateral velocity command by the operator. The asymmetric dynamic shared control determined the flow field’s rotational direction based on this intent, breaking the potential field symmetry. Figure 11d quantifies the longitudinal environmental constraint information perceived by the operator. During the 19–24 s interval, a negative peak of −0.19 N was observed in the longitudinal force feedback. Figure 11e quantifies the lateral environmental constraint information. During the 22–25 s interval, a negative peak of −0.18 N was observed in the lateral force feedback. Conflict Detection and Stiffness Arbitration Mechanism:

In Figure 12a, the stagnation index *λ_stagnation_* reached a peak at *t* = 20 s, corresponding to the moment when the formation began entering the narrow corridor and the vehicle decelerated for safe obstacle avoidance. In Figure 12b, the conflict index *λ_conflict_* accurately captured the physical constraint opposition during entry compression (*t* = 19 s) and exit expansion (*t* = 30 s). In Figure 12c, the deadlock index D*_lock_* did not reach the threshold throughout the entire narrow passage traversal. In Figure 12d, the arbitration factor *α* increased adaptively during the bottleneck passage, reaching a peak at *t* = 20 s to address the heightened conflict during configuration recovery.

**Figure 13:**
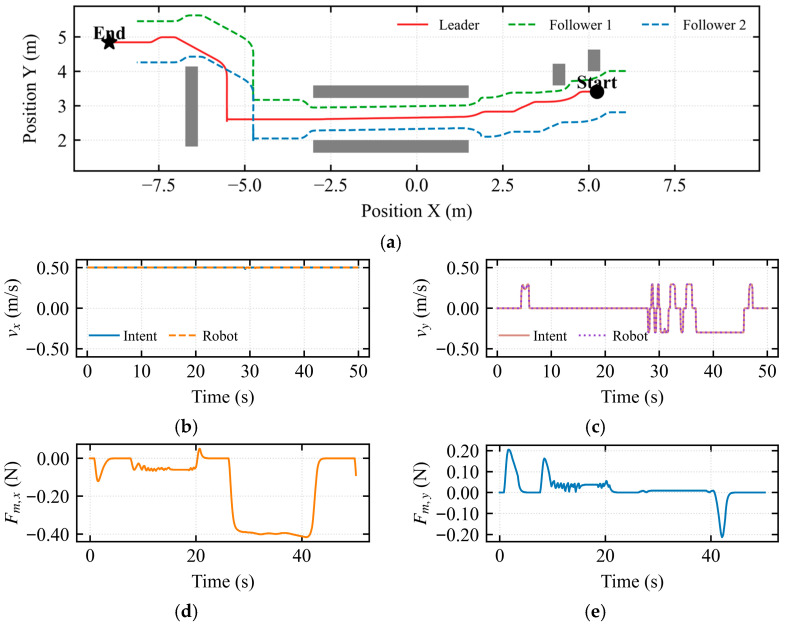
Conventional APF in the Narrow passage. (**a**) Narrow passage trajectory. (**b**) Longitudinal linear velocity. (**c**) Lateral velocity. (**d**) Longitudinal force. (**e**) Lateral force.

Figure 13a illustrates the trajectory under the APF method within a constrained narrow passage. Figure 13b,c illustrate the mapped longitudinal and lateral linear velocities from the haptic device, respectively. These results indicate that when encountering a longitudinal wall constraint, a continuous lateral velocity command is required to enable the robot’s escape, resulting in a prolonged traversal time. Figure 13d shows significant longitudinal force fluctuations around t=15s, indicating intermittent oscillations of the multi-robot formation along the motion direction. Furthermore, Figure 13e displays lateral force perturbations between 15s and 20s, revealing periodic lateral instability and swaying of the formation.

A comparative experiment was conducted between the traditional APF-based navigation and the proposed IM-AVM strategy in a narrow-channel scenario. Under traditional APF control, the multi-robot formation exhibited significant oscillations, characterized by longitudinal tremors before entering the passage and lateral instability while traversing the narrow space. Consequently, the completion time for the APF-governed formation was considerably longer than that of the IM-AVM strategy. Furthermore, when encountering long-wall obstacles post-passage, the traditional APF displayed high operator dependence; the formation frequently stagnated in local minima unless the human operator provided continuous, corrective commands. In contrast, the IM-AVM strategy required only a transient micro-intention force from the operator to break symmetry and navigate around the wall. Evidently, the proposed IM-AVM-based shared control framework demonstrates superior autonomy and robustness compared to traditional APF strategies.

### 5.4. Comprehensive Formation and Long-Distance Navigation

The robot formation was required to maintain a triangular configuration, navigate under operator guidance, avoid obstacles, and reach the destination.

**Figure 14:**
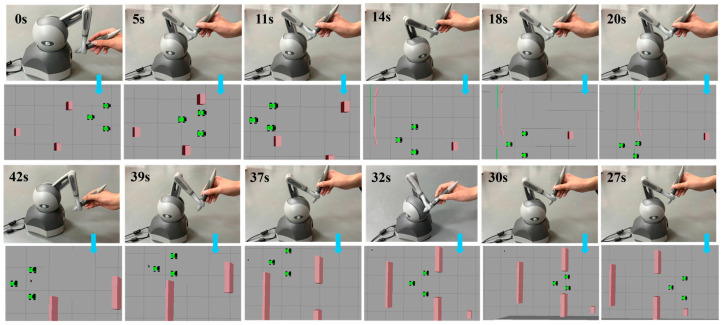
Snapshots of the master-slave collaborative formation teleoperation experimental process in a complex obstacle environment.

Figure 14 presents snapshots of the experimental process for master-slave collaborative formation teleoperation in a complex obstacle environment. The real-time poses and obstacle avoidance trajectories of the slave-side robot formation within the Gazebo environment are displayed, illustrating formation maintenance and trajectory tracking during obstacle avoidance. The master-side operator provided real-time control via a Geomagic Touch haptic device. Blue arrows indicate the temporal synchronization between the master-side operator’s hand intent and the slave-side formation response at different moments.

**Figure 15:**
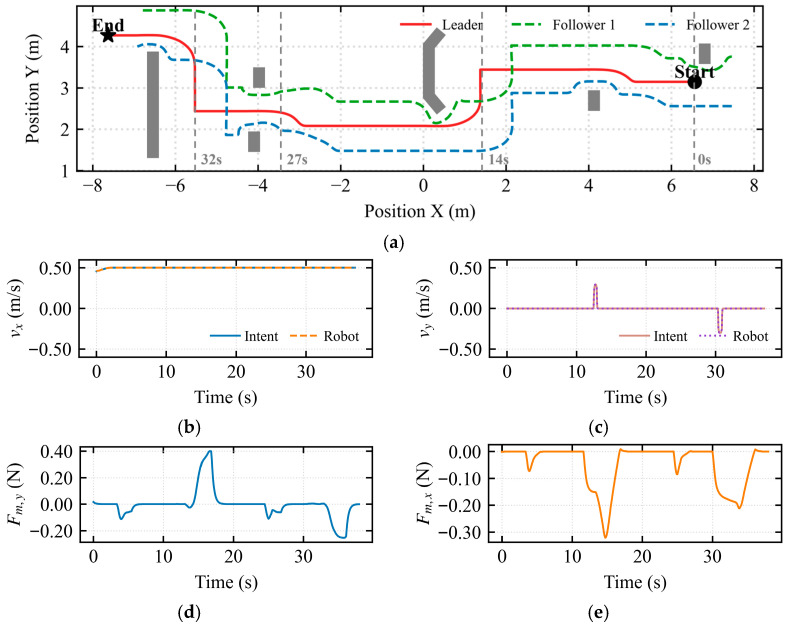
Formation trajectory in a comprehensive scenario. (**a**) Trajectory in Complex Scenarios. (**b**) Longitudinal linear velocity. (**c**) Lateral velocity. (**d**) Longitudinal force. (**e**) Lateral force.

As shown in Figure 15a, the robot formation exhibited collective obstacle avoidance and individual follower obstacle avoidance. Specifically, at positions *X* ≈ 1 m and *X* ≈ −5.5 m, the formation avoided a U-shaped opening and a long wall, respectively, through intent-mediated asymmetric shared control. Within the interval *X* ∈ [−5, −3], a safe tangential flow field was utilized for navigation through the narrow corridor. Throughout the formation process, the Adaptive Nonsingular Fast Terminal Sliding Mode Controller (ANFTSMC) reduced trajectory tracking oscillations, successfully maintained the topological connectivity of the formation, and prevented any instances of falling behind or collision. Figure 15b indicates that the mapped longitudinal velocity was consistently maintained at approximately 0.5 m/s. In Figure 15c, pulses of approximately 0.25 m/s and −0.25 m/s were observed near *t* ≈ 14 s and *t* ≈ 32 s, respectively, resulting from brief lateral velocity commands applied by the operator. This demonstrates that the intent-mediated asymmetric dynamic shared control leverages the operator’s micro-intentions to dynamically determine the flow field’s rotational direction, thereby breaking the potential field symmetry. Figure 15d reveals the active intervention mechanism of force feedback in longitudinal velocity. During high-risk obstacle avoidance moments, such as at *t* ≈ 14 s (U-shaped opening) and *t* ≈ 32 s (long wall), the longitudinal force *F_m__,__x_* exhibited negative peaks (approximately −0.32 N). This indicates that when an obstacle was detected ahead, the system actively applied a backward damping force to the master haptic device, counteracting the operator’s pushing action. This mechanism ensured safety and compliance of longitudinal operations within complex constrained spaces. Figure 15e illustrates that during the interval *t* ∈ [14, 18] s, a positive pulse with a magnitude of up to 0.4 N was generated in the lateral force, significantly reducing the operator cognitive load and steering errors during long-duration teleoperation tasks.

**Figure 16:**
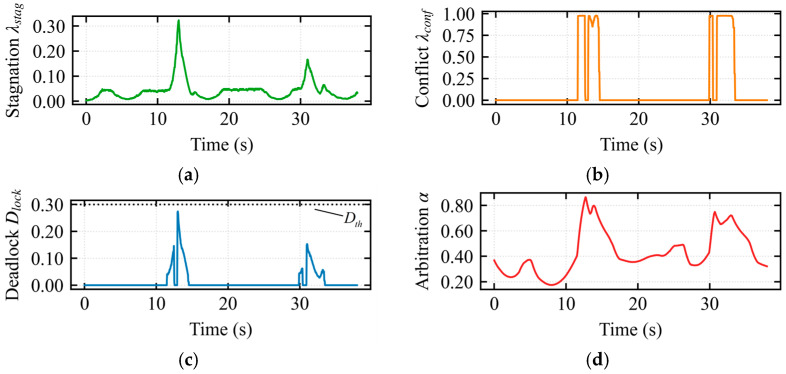
Formation trajectory in a comprehensive scenario. (**a**) Stagnation index *λ_stagnation_.* (**b**) Interaction conflict index *λ_conflict_.* (**c**) Deadlock index D*_lock_.* (**d**) Arbitration factor α.

In Figure 16a, the stagnation index *λ_stagnation_* reached a peak at *t* ≈ 13 s, corresponding to a period of continuous operator input with minimal robot displacement. In Figure 16b, the saturation of the conflict index D*_lock_* during the intervals *t* ∈ [11, 14] s and *t* ∈ [30, 34] s reflects the extreme geometric constraints encountered during obstacle avoidance at the U-shaped opening and long wall, respectively. In Figure 16c, the transient peaks of the deadlock index D*_lock_* indicate that the system remained in the assisted driving phase throughout. In Figure 16d, the arbitration factor α*α* increased significantly during the intervals *t* ∈ [11, 14] s and *t* ∈ [30, 34] s to cope with the challenges posed by the U-shaped opening and long wall.

**Figure 17:**
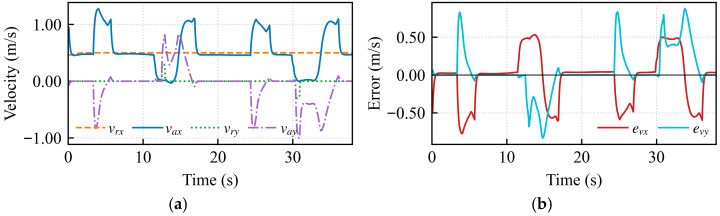
Trajectory tracking and tracking error of the leader. (**a**) Trajectory tracking. (**b**) Tracking error.

Figure 17a demonstrates the adaptive velocity regulation capability of the system in complex environments. The operator’s commanded velocity remained around 0.5 m/s. The actual velocity exhibited significant autonomous adjustment characteristics during obstacle avoidance (e.g., the U-shaped opening at *t* = 12−14 s and the long wall region at *t* = 30−32 s). The system prioritized safety by reducing the longitudinal velocity to near 0 m/s. Subsequently, during escape moments (e.g., around *t* = 15 s and *t* = 33 s), the actual velocity rapidly increased to approximately 1.2 m/s. This velocity compensation mechanism effectively mitigated the positional lag accumulated during obstacle avoidance.

Figure 17b further quantifies the velocity tracking error during this process. During obstacle avoidance maneuvers (around *t* = 12 s and *t* = 30 s), the robot was forced to decelerate while the reference trajectory continued to evolve, causing fluctuations in both longitudinal and lateral velocity errors, with peaks reaching approximately 0.5 m/s and 0.8 m/s, respectively. However, upon clearing the obstacle zone, the error curves converged back to zero within a short time, without exhibiting divergence or persistent oscillations. This validates the robustness and high dynamic response capability of the control algorithm even after experiencing significant nonlinear velocity interventions.

**Figure 18:**
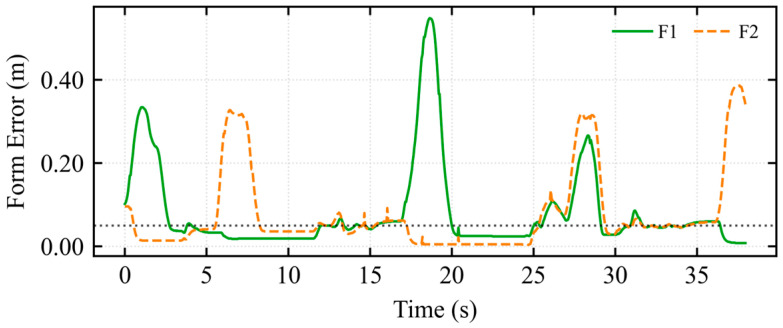
Trajectory tracking and tracking error of the leader.

Figure 18 illustrates the convergence characteristics of the formation control system when subjected to high dynamic disturbances. As observed from the error curves, when the leader executed aggressive maneuvers causing transient deviations in Follower 1 (F1) and Follower 2 (F2) at instances such as *t* ≈ 18 s for F1 and *t* ≈ 7 s and 28 s for F2, the curves exhibited a pronounced impulsive decay pattern. This phenomenon confirms the strong suppression capability of the adaptive terminal controller against nonlinear disturbances. Through real-time gain adjustment, the system state was rapidly restored from large deviations to a steady state within a finite time, thereby ensuring formation maintenance.

Further analysis of the error distribution reveals an asynchronous interleaving characteristic in the peak errors of F1 and F2 along the time axis. This precisely reflects the differentiated geometric and kinematic pressures experienced by individual robots within the multi-robot formation navigating a complex trajectory. Both the primary peaks of F1 and the secondary peaks of F2 correspond to the dynamic constraints imposed on each robot during specific curved path segments. Conversely, during non-maneuvering steady intervals (e.g., 10 s to 15 s), both followers consistently maintained extremely low error levels. This validates the robustness of the control algorithm at the multi-robot coordination level, guaranteeing high-precision formation maintenance under diverse operational conditions.

### 5.5. Scalability and Generalization to Multi-Robot Formations (n=3)

To address the potential scalability concerns, the experimental setup is expanded from a three-robot formation to a four-robot formation (n=3 followers).

**Figure 19:**
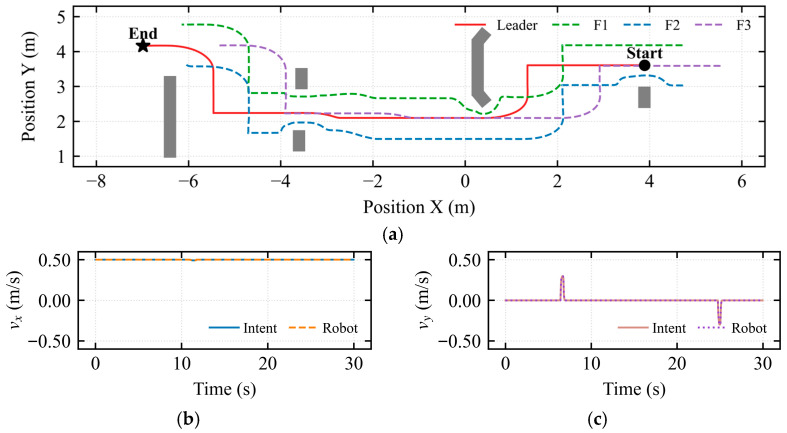

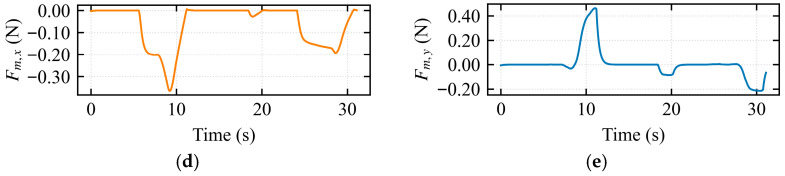
Multi-robot formation navigation in the generalized scenario (n=3). (**a**) Trajectory in Complex Scenarios. (**b**) Longitudinal linear velocity. (**c**) Lateral velocity. (**d**) Longitudinal force. (**e**) Lateral force.

Figure 19a illustrates the formation navigation consisting of one leader and three followers (n=3), which validates that the proposed shared control framework is seamlessly scalable to multiple followers. This demonstration confirms the intrinsic extensibility of both the intent-mediated asymmetric modulation (IM-AVM) strategy and the adaptive non-singular fast terminal sliding mode control (ANFTSMC). Figure 19b presents the mapped longitudinal linear velocity from the haptic master, while Figure 19c displays the mapped lateral linear velocity, Figure 19a,b present the Longitudinal force and Lateral velocity.

Although the distributed simulated platform employed in this study introduces real-world communication delays and human-interaction perturbations by integrating physical haptic hardware and communication networks, the dynamic evolution of the controlled objects remains based on the Gazebo physics engine (e.g., ODE solver). It must be acknowledged that non-linear slip of Mecanum wheels and irregular ground friction in real environments exhibit higher levels of stochasticity. Nevertheless, the simulated experimental framework utilized in this research can be regarded as a critical functional verification phase prior to physical deployment, establishing a logical foundation for subsequent migration to a full physical platform.

## 6. Conclusions

This study proposed and validated an integrated bilateral shared teleoperation framework for omnidirectional mobile robot formations. The unified modulation matrix (IM-AVM) successfully resolved the local minima problem inherent in traditional potential field methods. The eigenvalue-driven compliant compression mechanism addressed geometric mismatch challenge mathematically provable obstacle avoidance safety. The ANFTSMC ensures robust formation tracking under simulation settings, effectively handling modeled disturbances. This functional verification validates the control architecture and prepares it for future experimental transition to physical Mecanum-wheeled robots. High-frequency chattering was effectively suppressed using a saturation function, significantly improving trajectory tracking accuracy. The Smith predictor-based haptic feedback, while ensuring stability under time delays, provided the operator with clear, high-fidelity environmental geometric perception and haptic guidance. This framework not only enhances the operational capability of multi-robot systems in extreme environments but also offers a new theoretical paradigm for the design of future human–robot intelligent symbiotic systems: connecting autonomous planning and human perception through a unified mathematical description (the modulation matrix), Future research will extend the current translational mapping to include active macro-heading adaptation for navigating sharp L-shaped corners, and focus on conducting formal user studies—utilizing metrics such as the NASA-TLX and task completion time—to quantitatively evaluate the impact of the proposed framework on reducing operator cognitive workload.

## Figures and Tables

**Table 1:** Parameters for Intent-Mediated Avoidance and Haptic Feedback.

Parameter	Description	Value
η	Repulsion field gain	2.0
$\rho_{0}$	Obstacle influence radius	1.2 m
$\delta_{hyst}$	Hysteresis threshold	0.15
$D_{th}$	Deadlock composite index threshold	0.5
$T_{trig}$	Deadlock confirmation time	1.5 s
$K_{h}$	Haptic force feedback gain	1.0
$k_{guide}$	Guidance force scaling factor	0.25

**Table 2:** Parameters for NFTSMC Tracking and Formation Control.

Parameter	Description	Value
$c_{1}$	Linear sliding surface gain	2.0
$c_{2}$	Nonlinear sliding surface gain	1.0
p,q	Fractional power parameters	6, 5
μ	Robust switching gain	1.5
Φ	Boundary layer thickness	0.05
$\gamma_{x},\gamma_{y}$	Reaching law gains for X and Y axes	0.1, 0.1
$k_{p}$	Proportional gain for formation error	1.5
$W_{nom}$	Nominal passable width	1.6 m

## Data Availability

The original contributions presented in this study are included in the article. Further inquiries can be directed to the corresponding author.

## Abbreviations

The following abbreviations are used in this manuscript:

IM-AVM	Intent-Mediated Asymmetric Vortex Modulation	Multidisciplinary Digital Publishing Institute
ANFTSM	Adaptive Nonsingular Fast Terminal Sliding Mode Controller	Directory of open access journals
APF	Artificial Potential Field	
PD	Proportional-Derivative	Three letter acronym
TD	Tracking Differentiator	Linear dichroism
